# Reconstructing the evolutionary history of F_420_-dependent dehydrogenases

**DOI:** 10.1038/s41598-018-35590-2

**Published:** 2018-12-04

**Authors:** M. Laura Mascotti, Hemant Kumar, Quoc-Thai Nguyen, Maximiliano Juri Ayub, Marco W. Fraaije

**Affiliations:** 1 IMIBIO-SL CONICET, Facultad de Química Bioquímica y Farmacia, Universidad Nacional de San Luis, Ejército de los Andes 950, D5700HHW San Luis, Argentina; 2 Molecular Enzymology Group, University of Groningen, Nijenborgh 4, 9747 AG Groningen, The Netherlands; 3 Scuola Universitaria Superiore IUSS Pavia, Piazza della Vittoria 15, 27100 Pavia, Italy; 4 Faculty of Pharmacy, University of Medicine and Pharmacy, Ho Chi Minh City, 41 Dinh Tien Hoang Street, Ben Nghe Ward, District 1, Ho Chi Minh City, Vietnam

## Abstract

During the last decade the number of characterized F_420_-dependent enzymes has significantly increased. Many of these deazaflavoproteins share a TIM-barrel fold and are structurally related to FMN-dependent luciferases and monooxygenases. In this work, we traced the origin and evolutionary history of the F_420_-dependent enzymes within the luciferase-like superfamily. By a thorough phylogenetic analysis we inferred that the F_420_-dependent enzymes emerged from a FMN-dependent common ancestor. Furthermore, the data show that during evolution, the family of deazaflavoproteins split into two well-defined groups of enzymes: the F_420_-dependent dehydrogenases and the F_420_-dependent reductases. By such event, the dehydrogenases specialized in generating the reduced deazaflavin cofactor, while the reductases employ the reduced F_420_ for catalysis. Particularly, we focused on investigating the dehydrogenase subfamily and demonstrated that this group diversified into three types of dehydrogenases: the already known F_420_-dependent glucose-6-phosphate dehydrogenases, the F_420_-dependent alcohol dehydrogenases, and the sugar-6-phosphate dehydrogenases that were identified in this study. By reconstructing and experimentally characterizing ancestral and extant representatives of F_420_-dependent dehydrogenases, their biochemical properties were investigated and compared. We propose an evolutionary path for the emergence and diversification of the TIM-barrel fold F_420_-dependent dehydrogenases subfamily.

## Introduction

The naturally existing deazaflavin cofactor F_420_ is a peculiar cofactor involved in central metabolism of some specific Archaea and Bacteria lineages. It shows important structural differences compared to the ubiquitous FAD and FMN flavin cofactors. Compared with the canonical flavins, F_420_ has a hydroxyl group replacing the 8-methyl group, it lacks the 7-methyl group and, more interestingly, a C atom is replacing the N atom at the 5 position of the characteristic isoalloxazine ring. Furthermore, it contains an atypical group connected to the ribityl moiety through a phospholactyl linker: a poly-γ-glutamyl chain of varying length^1^. The redox potential of free F_420_ is −340 mV, much lower than that of free FAD and FMN and comparable to the nicotinamide cofactor NAD(P)H. Being a deazaflavin, the semiquinone form of F_420_ is highly unstable, making it an obligate hydride transferring cofactor^2,3^. While for a long time F_420_-dependent enzymes were considered as a rare enzyme class, research in recent years has surprisingly revealed that such enzymes are far more widespread and form a significant part of some bacterial and archaeal proteomes^4^.

F_420_ metabolism in methanogenic Archaea has been precisely described^5,6^. This cofactor plays a role in multiple central redox reactions such as the oxidation of energy sources and CO_2_ fixation. In bacteria, the physiological role of F_420_ is somewhat enigmatic. It has been proposed that F_420_ is an alternative hydride source to NAD(P)H that allows better control of the electron flow in redox reactions^7^. Different genera have been described to harbour F_420_-dependent enzymes, among them *Mycobacterium*, *Streptomyces*, *Nocardia* and *Chloroflexi*^8,9^. Undoubtedly, most research has focused on studying *Mycobacterium tuberculosis* F_420_-dependent enzymes which are involved in prodrug activation^10,11^. A significant amount of *M. tuberculosis* proteome is made of F_420_-dependent proteins. These are mainly distributed among three classes: luciferase-like monooxygenases (LLM), pyridoxamine 5′-phosphate oxidases (PPOX), and deazaflavin-dependent nitroreductases (DDN), most of them belonging to the LLM family^12^. Remarkably, all these classes also include FAD and FMN-dependent enzymes. Unfortunately, several classification criteria have been proposed for these enzymes and literature is difficult to bring together. Aflatoxin degrading F_420_-dependent reductases from *Mycobacterium smegmatis* were shown to belong to a class called F_420_-dependent reductases (FDR-A, FDR-B) which are related by sequence similarity to members of the PPOX family^13^. More recently, Ahmed *et al*., proposed that previously called FDRs should be referred to as flavin/deazaflavin oxidoreductases (FDORs A and B). FDOR A includes DDNs while FDOR B encompasses the so-called PPOX deazaflavoenzymes and enzymes using FMN, FAD and heme cofactors^14^. Although the three above-mentioned major deazaflavoprotein families are structurally distinct, there is a common pattern: they also include proteins that rely on other flavin cofactors or even non-related ones, such as heme or tetrahydromethanopterin (HMPT). This opens the question on what the evolutionary paths of these different families were and which constraints determined how the cofactor switching events could have occurred.

In this work, we aimed to describe the evolutionary events that gave rise to the F_420_-enzymes belonging to the luciferase-like class. Particularly, we focused on the members that act as dehydrogenases. By carefully describing the evolutionary history of dehydrogenases, we discovered a new class of enzymes and characterized two members from this group. In addition, to thoroughly understand the sequence of changes that led to the emergence of the different dehydrogenase functionalities, we reconstructed ancestral sequences and characterized an ancestral F_420_-dependent dehydrogenase.

## Results

### Structural clustering of F_420_-dependent enzymes

A relatively large number of F_420_-dependent enzymes are related to FMN-dependent luciferases by sequence similarity and structure, suggesting that they form a major family. Investigating the structural information reveals all these F_420_- and FMN-dependent enzymes share a TIM barrel fold and belong to the CATH 3.20.20.30 superfamily. While CATH classification typifies this superfamily as “FMN-dependent fluorescent proteins,” clearly it also has members that specifically bind a deazaflavin cofactor. Examples of some well-characterized FMN-dependent enzymes in this superfamily are the bacterial luciferases^15,16^ and the Type II Baeyer–Villiger monooxygenases^17^. On the other hand, some enzymes using F_420_ are the archaeal methylenetetrahydromethanopterin reductases (MERs)^18,19^ and bacterial glucose-6-phosphate dehydrogenases (FGDs)^20,21^. While at first sight it seems surprising to observe that this superfamily harbours enzymes using two different cofactors, FMN and F_420_ show quite some similarities. Both contain a phosphorylated riboflavin moiety which, in the case of F_420_ is slightly modified in the isoalloxazine part^1^. In addition, it has been shown that some F_420_-dependent reductases are also able to bind FMN and this modifies the enzyme reactivity^22^. To address the question on the cofactor divergence we propose that from ancestral FMN-dependent enzymes the deazaflavin cofactor specificity evolved, or *vice versa*. In this scenario, one aim of this study is to understand how such switch in cofactor dependence occurred.

### Evolutionary history of luciferase-like F_420_-dependent enzymes

**Figure 1:**
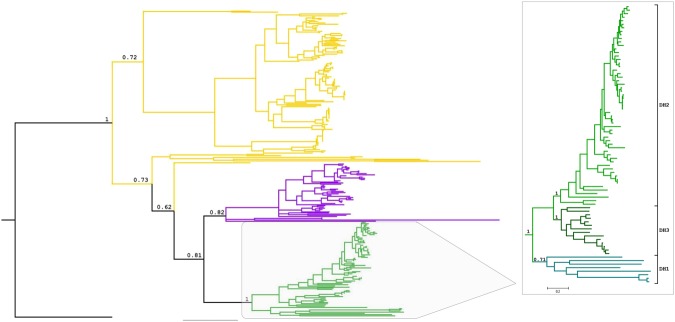
Phylogeny of the luciferase-like superfamily. Molecular phylogenetic analysis by Bayesian Inference from a MSA of full sequences. Posterior probabilities (PP) values corresponding to most important divergences are indicated above the branches. The sequence of an alanine racemase from *Thermaerobacter marianensis* (Uniprot code: E6SIZ8) was used as an external group to root the tree (black branch). The colour of the branches indicates: FMN-dependent enzymes (dark yellow), F_420_-dependent reductases (purple) and F_420_-dependent dehydrogenases (green). For a version of the tree including all PP values and taxa names please refer to SI. Right inset: Dehydrogenases tree. Monophyletic clades are coloured in different ranges of green: DH1 (bluish green), DH2 (green) and DH3 (dark green). PP values are indicated above the branches.

To understand the evolutionary relationships among the enzymes using F_420_ and FMN, a representative and non-redundant dataset was carefully constructed employing both structures and Hidden Markov Models (HMM) profiles in homology searches. Interestingly, a thorough phylogenetic analysis of the retrieved sequences shows that all the F_420_-dependent enzymes form a clade [posterior probability (PP) = 0.81] (Fig. 1 and S1, Data S1). This indicates that the F_420_-enzymes of this superfamily have evolved from a single ancestral protein, probably by the accumulation of major changes in the cofactor binding pocket.

Inside the lineage of F_420_-dependent enzymes two clades are observed: one including the so-called MERs (PP = 0.82) and the other gathering the FGDs and F_420_-alcohol dehydrogenases (PP = 1). Therefore, these two clades will be called from now on *reductase* and *dehydrogenase* groups, respectively. In the reductase group a few divergent sequences are observed: some archaeal uncharacterized sequences and the phthiodiolone/phenolphthiodiolone dimycocerosates ketoreductase from *Mycobacterium bovis* (Uniprot ID: Q7TXK4)^23^. The latter enzyme was recently shown to act as a F_420_-dependent reductase reducing phthiodiolones to phthiotriols^24^, in line with other members of this group utilizing F_420_H_2_. In the dehydrogenases clade, three subgroups are observed: DH1 (PP = 0.71), DH2 (PP = 1), and DH3 (PP = 1) (inset Fig. 1). The most basal group of sequences form the DH1 clade and include the unique dehydrogenases: Adf from *Methanoculleus thermophilus* (Uniprot ID: O93734, PDB: 1RHC), catalysing oxidation of small aliphatic alcohols^25^, and FGD2 from *M. tuberculosis* (Uniprot ID: P96809), which catalyses the oxidation of hydroxymycolic acid to ketomycolic acid^26^. Also some other uncharacterized archaeal sequences are found. The two other well-defined subgroups, DH2 and DH3, can be recognized as: DH2 contains the already characterized FGD from *M. tuberculosis* (Uniprot ID: P9WNE1, PDB: 3B4Y)^20^ and *Rhodococcus jostii* RHA1 (Uniprot ID: Q0RVH7, PDB: 5LXE)^21^ and DH3 contains non-characterized proteins from various bacterium species.

### Experimental characterization of the newly identified dehydrogenases clade

As it was described before, the clade DH3 in the dehydrogenases family is formed by uncharacterized bacterial sequences. To explore the characteristics of members of this group, two sequences were selected for experimental characterization: FGD-Noca from *Nocardioidaceae bacterium* (GenBank: EGD40158.1) and FGD-Cryar from *Cryptosporangium arvum* (GenBank: WP_035860858.1). The FGD-Noca and FGD-Cryar sequences showed 59 and 37% identity (>90% coverage) to mycobacterial characterized FGDs, respectively, and 71% identity between them. Both enzymes could be overexpressed as soluble proteins in *Escherichia coli* either as native protein (FGD-Noca) or as a fusion protein with the partner SUMO (FGD-Cryar) (Figure S2). The proteins were purified and their enzymatic properties were investigated. As the closest known homologs are true FGDs, both putative dehydrogenases were first assayed for FGD activity. This revealed that they can oxidize d-glucose-6-phosphate with significant activity. Yet, interestingly, it was found they exhibit a broader substrate acceptance than all previously characterized FGDs, which are rather specific towards d-glucose-6-phosphate. Other 6-phosphate sugars were also well accepted by both DH3 dehydrogenases (Table S1).

**Figure 2:**
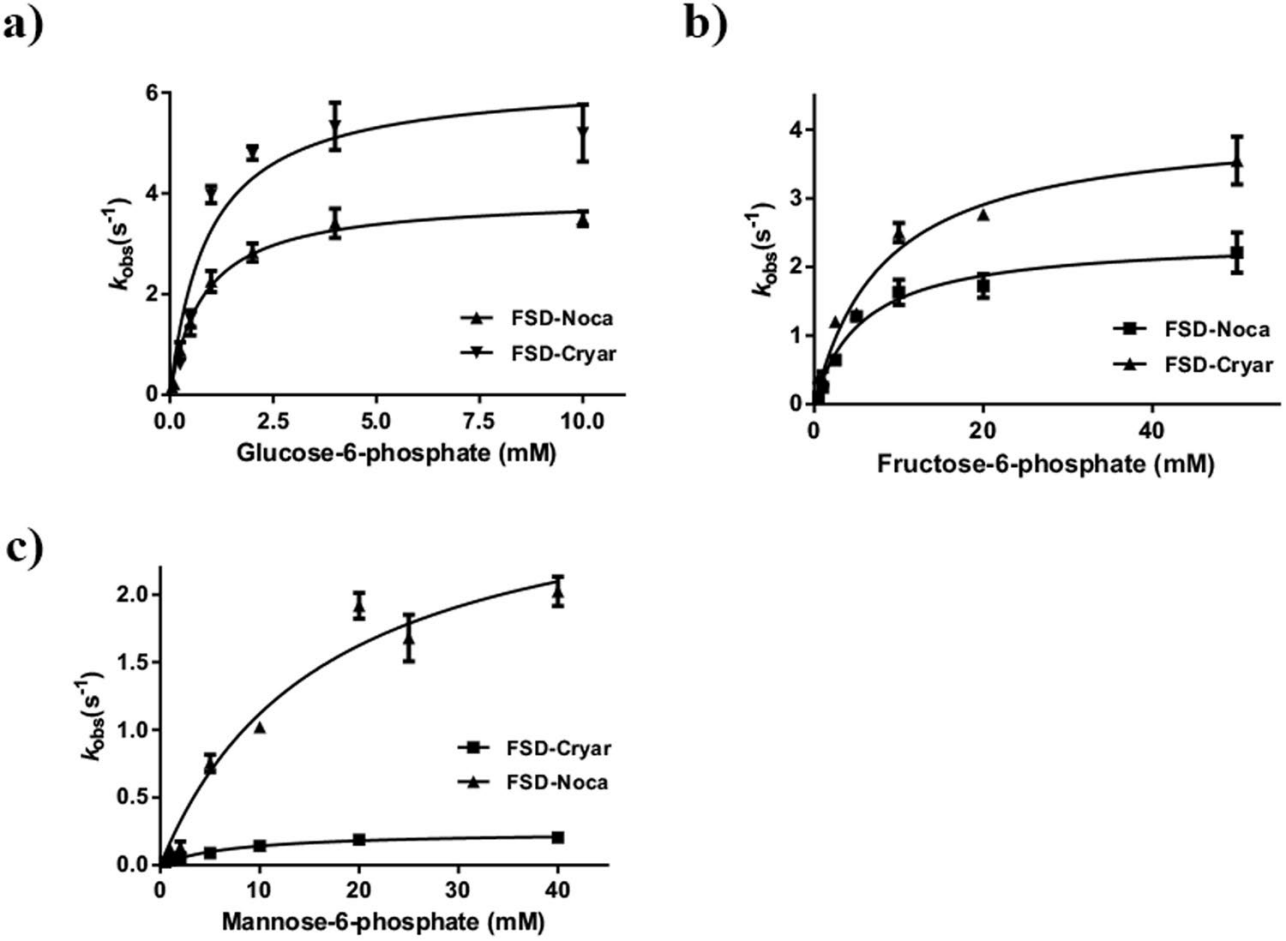
Steady-state kinetics of FSDs. Steady-state kinetic experiments were conducted following the reduction of F_420_ (ε_400_ = 25.7 mM^−1^cm^−1^). Three different substrates were employed: (**a**) d-glucose-6-phosphate, (**b**) d-fructose-6-phosphate, (**c**) d-mannose-6-phosphate.

**Figure 3:**
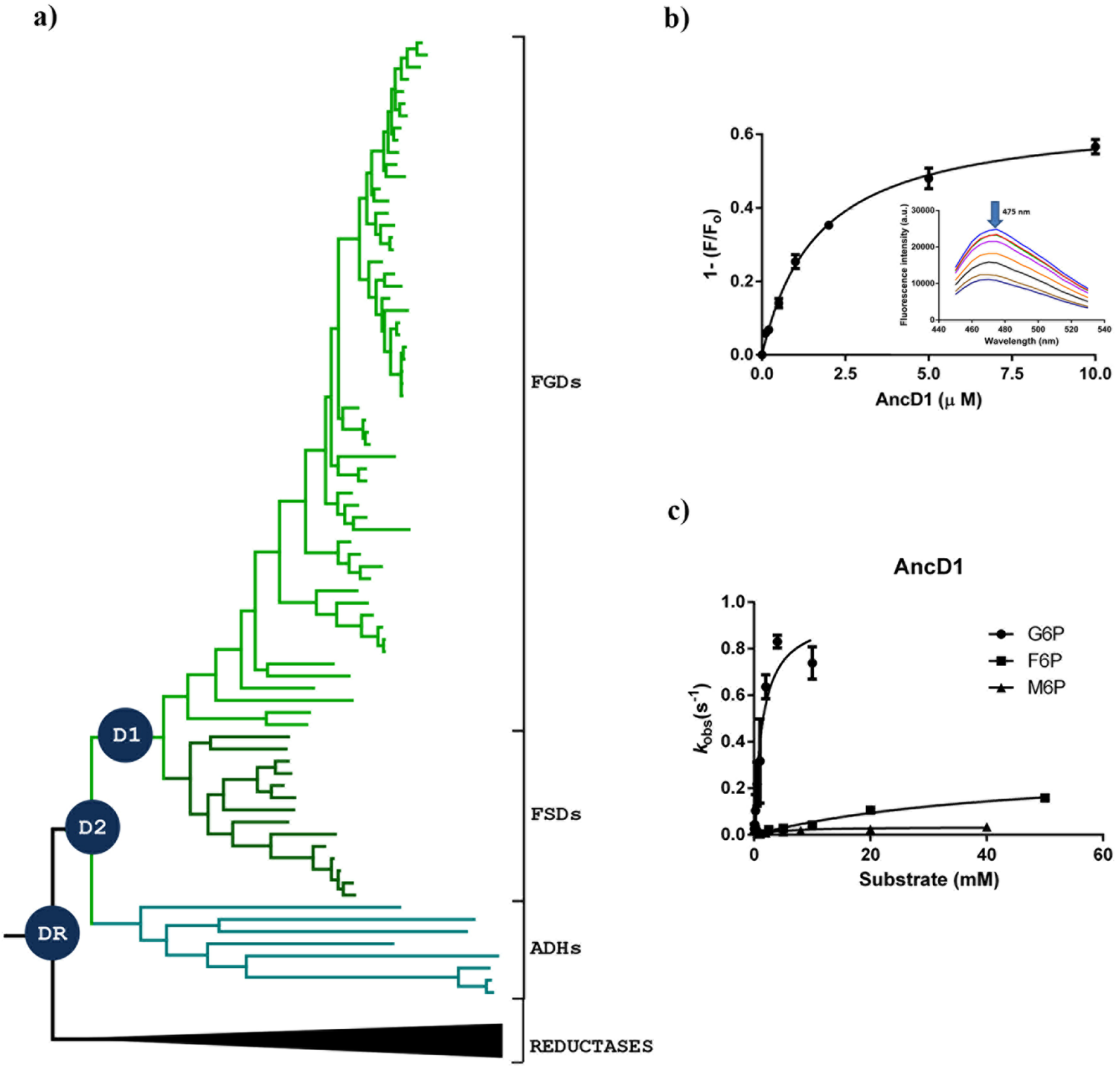
Ancestral sequence reconstruction of dehydrogenases subfamily. (**a**) Dehydrogenases tree with reconstructed ancestral nodes. Groups are presented as FGDs, FSDs and ADHs. Blue circles represent selected nodes for experimental characterization: DR (Ancestor of Dehydrogenases and Reductases), D2 (Cenancestor of FGDs, FSDs and ADHs) and D1 (Ancestor of FGDs and FSDs). (**b**) Binding of AncD1 to F_420._ Fluorescence spectrum was measured using excitation and emission wavelengths at 420 nm and 475 nm, respectively. (**c**) Kinetic features of AncD1 enzyme. Steady-state kinetic experiments were conducted following the reduction of F_420_ (ε_400_ = 25.7 mM^−1^cm^−1^).

The observed substrate scope and kinetic profiles are in stark contrast with the typical FGDs (Table 1 and Fig. 2). FGD-Noca displayed high affinity not only for d-glucose-6-phosphate (G6P) (*K*_M_ = 0.94 mM) but also for d-fructose-6-phosphate (*K*_M_ = 4.5 mM). Similarly, FGD-Cryar has a low *K*_M_ value for G6P (0.9 mM) and also good affinity for d-fructose-6-phosphate (*K*_M_ = 6.1 mM) and d-mannose-6-phosphate (*K*_M_ = 7 mM). FGD-Msmeg from *M. smegmatis* transforms exclusively G6P (*K*_M_ = 1.6 mM) and no activity is observed with other phosphorylated sugars^27^, while FGD-Mtb from *M. tuberculosis* behaves similarly (*K*_M_,_G6P_ = 0.1 mM)^20^. Likewise, FGD-Rha1 from *R. jostii* RHA1 is highly specific for G6P (*K*_M_ = 0.31 mM): <2% of other 6-phosphate 6-membered sugars are transformed by the enzyme when 10 mM of these substrates is employed compared to 1.0 mM of G6P^21^. Furthermore, FGD-Noca and FGD-Cryar could accept also non-phosphorylated 5- and 6-carbon sugars at high concentrations (400 mM) albeit with low rates. Based on these unusual features we called this clade FSDs, accounting for “F_420_ sugar-6-phosphate dehydrogenases”. From now on, we will refer to the FSDs described above as FSD-Noca and FSD-Cryar. Hence, the F_420_ dehydrogenases family has diverged into three subfamilies: alcohol dehydrogenases (ADHs), sugar-6-phosphate dehydrogenases (FSDs) and glucose-6-phosphate dehydrogenases (FGDs) (Fig. 3a).

**Table 1:** Biochemical characterization of extant (FSD-Noca and FSD-Cryar) and ancestral (AncD1) recombinant enzymes.

Substrates	FGD-Noca (FSD-Noca)		FGD-Cryar (FSD-Cryar)		AncD1	
	*k*_cat_ [s^−1^]	*K*_M_ [mM]	*k*_cat_ [s^−1^]	*K*_M_ [mM]	*k*_cat_ [s^−1^]	*K*_M_ [mM]
d-glucose-6-phosphate	4.1 ± 0.24	0.94 ± 0.19	6.2 ± 0.82	0.9 ± 0.4	0.9 ± 0.05	1 ± 0.19
d-fructose-6-phosphate	2.1 ± 0.2	4.5 ± 1.1	3.6 ± 0.41	6.1 ± 1.7	0.3 ± 0.05	45 ± 15
d-mannose-6-phosphate	2.6 ± 0.15	16.4 ± 2.5	0.23 ± 0.03	7 ± 2.7	0.04 ± 0.01	7.2 ± 4.7
d-glucose	—	>500	—	>500	—	>500
**Substrates [400 mM]**	***k***_**obs**_ **[s** ^**−1**^ **]**		***k***_**obs**_ **[s** ^**−1**^ **]**		***k***_**obs**_ **[s** ^**−1**^ **]**	
d-fructose	—		—		—	
d-mannose	0.02 ± 0.01		—		0.1 ± 0.03	
d-xylose	0.11 ± 0.05		0.13 ± 0.1		—	
**Binding affinity**	***K***_**D**_ **[µM]**		***K***_**D**_ [**µM]**		***K***_**D**_ [**µM]**	
F_420_	0.09 ± 0.02		2.2 ± 0.7		1.6 ± 0.1	
FMN	>150		>150		>150	
**Activity features**						
pH	6.5		7.5		6.5	
*T*_*m*_ [°C]	45.5		43		53

### Reconstruction of dehydrogenases ancestors

The evolution of the dehydrogenases was explored by performing ancestral sequence reconstruction in the quest of tracing the emergence of the sugar dehydrogenase functionality. Initially, a curated phylogeny was employed, including reductases as an external group. Also, a phylogeny containing only dehydrogenases (Data S2 and S3) was used and both outputs were compared. Three specific ancestral states were selected for further analysis: the node between the dehydrogenases and reductases (named AncDR), the cenancestor of the three dehydrogenases subfamilies’ (AncD2) and the ancestor shared by FSDs and FGDs (AncD1) (Fig. 3a). The accuracy of the reconstruction was low at the most divergent nodes, DR and D2, as expected (Figure S5). In case of ancestral sequence D1, although some ambiguous positions (20/340 with PP ≤ 0.7) were detected, alternative amino acids appeared as conservative changes. Therefore we decided to opt for the residues displaying the highest probability for gene synthesis.

### Experimental resurrection of the ancestral sugar dehydrogenase enzyme

Ancestral state D1 was successfully expressed in *E. coli* as a SUMO fusion protein (Figure S2). Although several expression strategies were assessed, no functional expression of AncDR and AncD2 was obtained. Purified AncD1 was found to tightly bind F_420_ (*K*_D_ = 1.5 µM, Fig. 3b) and displayed a substrate profile similar to that of FSDs (Table S1). Somewhat lower *k*_cat_ and higher *K*_M_ values were obtained compared to FGDs and FSDs (Table 1 and Fig. 3c). This suggests that, different from FGDs, the ancestral dehydrogenase D1 behaves as a more generalist enzyme which has not a much defined substrate preference.

**Figure 4:**
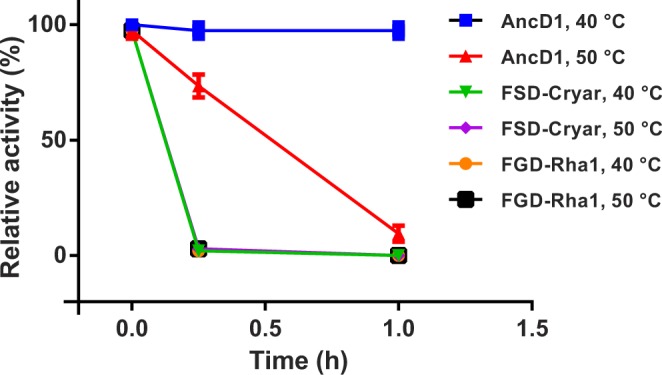
Thermal stability of FSD-Cryar, FGD-Rha1 and AncD1. Enzymes were incubated at 40 °C or 50 °C for 1 h and samples were collected after regular intervals (15, 30 and 60 minutes) for activity measurements.

Remarkably, when its melting temperature (*T*_m_) was assessed, AncD1 displayed a 10 °C higher *T*_m_ value (53 °C) than that of the studied FSDs and FGDs (≈43 °C), revealing that it is a rather thermostable enzyme. Moreover, upon incubating the enzyme at 40 °C and 50 °C, it was found that AncD1 retains almost full activity at 40 °C up to an hour while FSD-Cryar and FGD-Rha1 are totally inactive after a few minutes. Even more, at 50 °C AncD1 retained almost 80% of activity after 15 minutes of incubation (Fig. 4). These results clearly show that the resurrected AncD1 enzyme is much more robust than the modern FGDs and FSDs.

## Discussion

F_420_ is at the same time a versatile and exceptional cofactor in Nature. Its low redox potential makes it perfect to be involved either in reductions or oxidations^4^. Besides, its distribution seems restricted in taxonomy (frequent only in some Bacteria and a few Archaea groups) but not in functionalities. Various kinds of oxidoreductases have been described employing this deazaflavin coenzyme in combination, or not, with other cofactors^14^. When vetting structural databases searching for F_420_-dependent enzymes, the luciferase-like group appears particularly rich in these deazaflavoenzymes. This superfamily includes FMN- and F_420_-depending enzymes. Among these, those employing the deazaflavin cofactor perform opposite reactions: reductions or oxidations. These peculiarities invited us to rationalize the question on the origin of cofactor dependence divergence (FMN/F_420_), and the nature of the family’s common ancestor.

By inferring a rooted phylogeny we postulate the dependence of F_420_ emerged from an FMN-using ancestor, in a singular event, suggested by the well supported monophyly of the F_420_ clade (PP = 0.81) (Fig. 1). Closest FMN extant proteins include enzymes such as bacterial luciferases and type II BVMOs. The F_420_ family is clearly split into two clades, reductases and dehydrogenases, both including sequences from Bacteria and Archaea domains. The absence of members from Eukarya suggests that the copy of the ancestral gene originating the family might have been lost in this lineage. This type of taxonomic distribution is a common pattern when analysing non-essential gene families, as genetic drift is a major evolutionary driver^28,29^. Also, this indicates that the evolutionary events leading to the switch in cofactor usage occurred in primitive times (more than 4 bya), when the three domains of life were not yet defined^30,31^.

**Figure 5:**
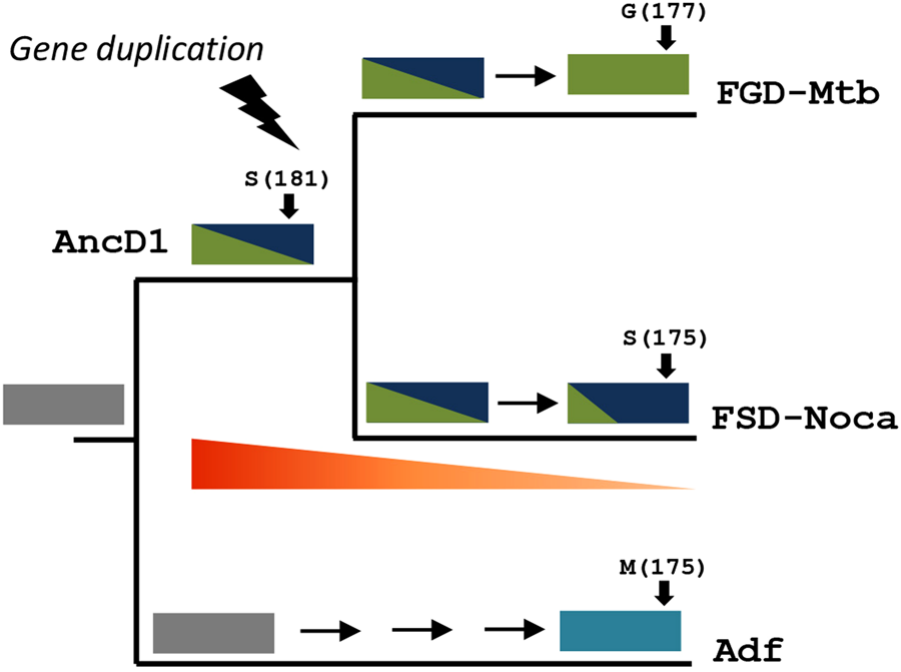
Evolutionary history of the clade FGD/FSD. A schematic tree is presented displaying in the tip of branches a representative enzyme of each class: FGD-Mtb from the FGDs (UniProt: P9WNE1), FSD-Noca (UniProt: E9V3D1) from FSDs and Adf (UniProt: O93734) from ADHs. Blocks represent genes and colours indicate their functionalities; green = G6P dehydrogenase, dark blue = sugar-6-phosphate dehydrogenase, cyan = secondary alcohol dehydrogenase, grey = unknown. Below the clade FGD/FSD a triangle in red to orange gradient symbolizes the decrease of thermostability observed from AncD1 to modern enzymes.

Focusing on the phylogeny of the dehydrogenases family, evolutionary history indicates the divergence into three subfamilies. The first emerging group includes the alcohol dehydrogenases as Adf and FGD2. These enzymes have been described to transform exclusively linear alcohols into ketones, while not accepting sugars as substrates^32,33^. After this early divergence, the emergence of two other groups is observed; one including the well-known FGDs and the other is described here by us, containing the FSDs. This newly characterized FSD clade is formed by enzymes displaying broader sugar acceptance profiles when compared to FGDs. Interestingly, when the taxonomic distribution of FGDs and FSDs was analysed, it was observed that some orders harbour both kinds of enzymes, such as Micrococcales (*e.g*. *Microbacterium* spp) and Propionibacteriales (*Nocardioides* spp), while other orders, such as the Corynebacteriales including the well-known *Mycobacterium* species, exclusively contain FGD-encoding genes (Table S2 and Figure S6). This scenario can be interpreted as that the FGDs arose through a functional optimization or subfunctionalization process from the duplication of an FSD like ancestral gene. To test this hypothesis we investigated when the sugar dehydrogenase functionality emerged. By resurrecting and experimentally characterizing the node before the divergence of FGDs and FSDs that interrogation could be solved. The resurrected ancestral enzyme (AncD1) was found to prefer sugars over linear alcohols with low specificity and affinity. Figure 5 shows how this promiscuous ancestral enzyme with relatively low activity evolved to present day dehydrogenases which display high activity for certain substrates and little or no activity for others. The emergence of the sugar oxidation function dates at least 3069 mya, accounting for the divergence of Actinobacteria and Chloroflexi phyla (Figure S6)^34^.

To understand the observed differences in substrate acceptance, a 3D model of AncD1 was constructed and compared to FSDs models, and FGD (3B4Y and 5LXE) and Adf (1RHC) structures. Although among FGDs and FSDs we expected differences in residues involved in the phosphate moiety recognition^20,21^, these were not found. In fact, differences in these residues were only found in comparison to Adf, as expected (mainly changes in Leu256/Cys249)^25^. Also, all other key residues described forming hydrogen bonds with the pyridine ring or involved in the hydride transfer mechanism were conserved^35^. However, major differences in the dimensions of the active site cavity were observed when analysing the structures. The Adf active site is very narrow compared to both FGDs and FSDs. Interestingly, this cavity is larger in AncD1, probably explaining its relaxed substrate specificity and suggesting that a constraint in the emergence of this enzyme lineage may have been imposed by the availability of more voluminous substrates (Figure S7). When the first layer of residues defining the substrate pocket was inspected (10 Å from the surroundings of the cofactor), we observed the conserved Gly177 from FGDs (numbering from 3B4Y) is replaced by a Ser (175 in FSD-Noca) in FSDs while in Adf this position is occupied by a more bulky Met (175). Interestingly, by analysing the evolutionary trajectory of this position we observed a first switching from Met to Ser (Adf → AncD1), as the sequence AncD1 displays a serine (PP = 0.99). Then, while FSDs conserved this serine, it changed to Gly in FGDs lineage probably influencing the exacerbated activity of this group towards G6P. Although deeper structural analyses are required, this might be the tip of the iceberg to disclose the molecular basis for substrate recognition^36^.

Finally and remarkably, we found that AncD1 is not only a generalist enzyme due to its catalytic properties, but also a very robust enzyme displaying 10 °C higher melting temperature compared to modern FGDs and FSDs. Even more, we found that AncD1 retains > 80% activity upon incubation at 50 °C. This thermostability trend has been observed for other resurrected enzymes, in agreement with the theory that protein stability must be sacrificed to support the conformational flexibility necessary for enzymatic activity^37,38^.

Based on our evidences we propose the evolutionary history of the F_420_-dependent dehydrogenases family has gone through, among other mechanisms, gene duplication followed by subfunctionalization, leading to more specific enzymes over time (Fig. 5). This history is in agreement with the EAC (escape of adaptive conflict) scenario when gene duplication takes place^39^. FSDs and FGDs evolved from the multifunctional AncD1 by the accumulation of changes, conferring both paralogs different subfunctions. While the first enzymes in this family were able to reduce simple linear alcohols, the use of sugars as substrates emerged as a new functionality later at a preduplication stage. After this, a gene duplication occurred and resulting paralogs display different subfunctions: a group of enzymes retained the ability of transforming a broader sugars’ profile (FSDs) while the other specific group evolved by functional optimization toward the most abundant sugar in biological systems, glucose (the FGDs). Our study provides a strong basis for future work on the discovery of novel F_420_ dehydrogenases and the engineering of available ones for biotechnological purposes, such as switching substrate specificities or enhancing thermal stabilities.

## Methods

### Dataset construction and evolutionary analyses

To identify the major F_420_-dependent enzyme superfamilies sequences of structure solved F_420_-using enzymes were collected from PDBsum and clustered by CATH^40^. Each superfamily was defined as sharing the four-numbered CATH code. Clustering was refined and reinforced by using the profile databases INTERPRO and Pfam^41,42^.

Luciferase-like superfamily dataset, including FMN- and F_420_-depending enzymes, was constructed by HMM-profiling search in reference proteomes and UniprotKB databases employing protein alignments in HMMER3^43^. For these searches, multiple sequence alignments (MSAs) of experimentally characterized enzymes were obtained in MAFFT v.7. Initially, 250 first hits (E ≤ 0.03) were collected. The generated HMM profile was used as input for a new search and the obtained 250 first hits (E ≤ 0.03) were also collected. This searching strategy was repeated restricting the taxonomy to each of the three domains life in order to vet all possible homologous sequences. All retrieved sequences were gathered, MSAs constructed and redundancy removed. Sequence annotated by structure (SAS) tool^44^ and ConSurf server^45^ were employed to characterize the HMM profiles of subfamilies inside the superfamily.

Phylogenetic analyses were performed employing Bayesian inference (Mr. Bayes v.3.2.6) with a mixed model until reaching convergence (1.500.000–2.000.000 generations, split frequency < 0.02). Maximum likelihood inference method was also implemented (PhyML v.3.0) with 500 bootstraps. Best fit model parameters were determined by the Akaike information criterion (ProtTest v.3.4). Rooting was performed by using the external group strategy, which was selected on the basis of structural homology as previously described^46^.

### Ancestral Sequence Reconstruction

Ancestral sequence reconstruction was performed using the maximum likelihood inference method (PAMLX software v.4.9). Sequences were analysed using an empirical amino acid substitution model (model = 3), fixed alpha = 0.607, 4 gamma categories and amino acid distance matrix G1974 (aadist = 1). The posterior probability distribution of ancestral states at each site was analysed at nodes corresponding to AncDR, AncD2 and AncD1 sequences. Sites were considered ambiguously reconstructed if the most likely state had a posterior probability < 0.7^47^. Sequences of targeted nodes were submitted to the Swiss-Model server to obtain 3D homology models. Structures were visualized, compared, and analysed using the PyMOL v.1.7.6 molecular visualization system and the Xtal-Pred web server^48^ was used to estimate the stability parameters.

### Expression and purification of ancestral and extant dehydrogenases

Genes with optimized codons for protein expression in *E. coli* were ordered from Thermo scientific and cloned into a pBAD vector (Invitrogen). Two expression vectors were generated: one for expressing the protein with a N-terminal 6 × His tag, while the other version resulted in expression of the target protein with a N-terminal 6 × His-SUMO tag. *Nde*I and *Hind*III restriction sites were used for cloning the pBAD-*fgd* constructs while *Nco*I and *Hind*III sites were used to make the pBAD-SUMO-*fgd* constructs. All constructs were confirmed by sequencing at GATC Biotech.

Plasmids were transformed into *E. coli* NEB^®^ 10-beta chemical competent cells for storage and expression. Overnight cultures of transformants were diluted 100 times in fresh 5 mL Terrific broth containing 50 µg/mL ampicillin and grown at 37 °C until OD_600_ reaches 0.6. Cells were then induced using 0.02% (w/v) of l-arabinose and further incubated at different temperatures (17, 24, 30 and 37 °C) to test expression. Constructs that resulted in expression of the target protein were used for growing large cultures and subsequent protein purification.

AncD1 and FSD-Cryar were expressed as SUMO fusion proteins while FSD-Noca was expressed as native protein. Proteins were expressed in *E. coli* TOP10 cells grown in Terrific broth containing 50 µg/mL ampicillin. Expression was induced by adding 0.02% (w/v) l-arabinose when cells reached an OD_600_ of 0.4–0.6 followed by incubation at 24 °C for 36 h while shaking at 200 rpm. Cells were harvested by centrifugation at 5500 × *g* for 15 min (Beckman–Coulter JA-9.1 rotor, 4 °C) followed by one washing step. Cells were re-suspended in lysis buffer containing 50 mM potassium phosphate (KPi) pH 7.5, 10% (v/v) glycerol, 1.0 mM β-mercaptoethanol, DNaseI (5 µg/mL) and disrupted by sonication (VCX130 Vibra-Cell, Sonics & Materials, Inc., Newtown, USA) with 10 sec on and 15 sec off cycles at 4 °C. This was followed by centrifugation at 15,000 × *g* (Beckman–Coulter JA-17 rotor, 4 °C) to remove cell debris.

Cell extract containing FGD-Noca was loaded on a 5 mL anion exchange column (Hi-Trap™ QFF) pre-equilibrated with buffer A [50 mM KPi pH 7.5, 10% (v/v) glycerol, 1.0 mM β-mercaptoethanol] using an FPLC (Aktapure, GE healthcare). Unbound proteins were removed by washing the column with the same buffer. The protein eluted at 20% of buffer B (1 M NaCl in buffer A) after running a linear gradient. AncD1 enzyme and extant FGD-Cryar were purified using TALON^®^ metal affinity resins. After equilibrating the resins with 5.0 mM of imidazole in buffer A, cell free extracts were incubated with the pre-equilibrated resins in a rocking shaker for 1 h at 4 °C. After incubation, the suspension was loaded into gravity flow columns and unbound proteins were let flow through. Then, the resin was washed with 10 column volume (CV) of the washing buffer [50 mM KPi pH 7.5, 10% (v/v) glycerol, 1.0 mM β-mercaptoethanol, 15 mM imidazole]. The target protein was eluted using 10 CV of the elution buffer [50 mM KPi pH 7.5, 10% (v/v) glycerol, 1.0 mM β-mercaptoethanol, 500 mM imidazole]. Purity of proteins was analysed by SDS-PAGE.

### Enzyme characterization

#### Substrate acceptance profiling

Sugars (phosphorylated as well as non-phosphorylated) and alcohols were tested as substrates. d-glucose-6-phosphate (G6P) was used as a prototype substrate to initially verify activity. Other 6-phosphorylated sugars tested were: d-fructose-6-phosphate, d-mannose-6-phosphate and d-glucosamine-6-phosphate. d-glucose, d-fructose, d-mannose, d-xylose, d-galactose-1-phosphate, d-glucose-1-phosphate, isopropanol, isobutanol, butanol and cyclohexanol were also tested for activity. Kinetics of all enzymes was measured in a Synergy MX microplate reader (BioTek) using 96-well F-bottom plates (Greiner Bio-One GmbH) at 25 °C. Assays were performed in a volume of 200 µL, containing 20 µM F_420_, 50–100 nM of enzyme and varying concentrations of the substrates in buffer (50 mM KPi, pH 7.5). Reaction was started by adding 100 µL of enzyme. All measurements were followed at λ = 400 nm for 3 min. The observed rates (*k*_obs_) were calculated by using a molecular extinction coefficient of ε_400_ (F_420_) = 25.7 mM^−1^cm^−1^. All experiments were repeated three times.

#### Binding assay

*K*_D_ values were determined based on the fluorescence quenching when the cofactor binds to the protein. A Synergy MX microplate reader (BioTek) with 96-well F-bottom black plates (Greiner Bio-One GmbH) at 25 °C was used for the measurements. For F_420_, an excitation wavelength of 420 nm and an emission wavelength of 475 nm were used, while FMN was excited at 450 nm and emission was recorded at 530 nm. A total reaction volume of 200 µL contained 0.1 µM of cofactor (F_420_ or FMN) and different concentrations of the protein in each well. The decrease in the fluorescence was plotted against the concentrations to obtain apparent *K*_D_ values. One site specific binding function of GraphPad Prism 6 software (version 6.04): F_obs_ = F_max_ * X/ (*K*_D_ + X), where F_obs_ is the observed fluorescence, and F_max_ is the fluorescence at a saturating concentration of the ligand, was used for plotting and calculating *K*_D_ values. Experiments where performed in duplicate.

#### Melting temperature and pH optimum

The apparent melting temperatures (*T*_m_) of studied proteins were determined using the Thermoflour^®^ technique with a Bio-Rad C1000 Touch Thermal Cycler (Bio-Rad Laboratories, Inc.). The reaction volume of 25 µL contained 10 µM of enzyme and 5 µL of 5 × SYPRO Orange (Invitrogen). Thermal stability was assayed by incubating the enzymes at 40 °C and 50 °C for 1 h and collecting samples every 15 min, followed by activity assay. As reference FGD-Rha from *R. jostii* RHA1 was employed^21^.

Enzyme activity at different pH values was measured by using 1 mM d-glucose-6-phosphate as substrate and 50 mM of buffer: sodium acetate (pH 4.5–5.5), potassium phosphate (pH 6.0–7.5) and tricine–KOH (pH 8.0–9.5).

## Electronic supplementary material

Supporting Information

## Data Availability

All data generated during this study are included in this published article and its Supplementary Information files.
