# Supplementary material for: Reconstructing the evolutionary history of F420-dependent dehydrogenases
Source: Sci Rep. 2018 Dec 4;8:17571. doi: 10.1038/s41598-018-35590-2 (PMC6279831; doi:10.1038/s41598-018-35590-2)
Supplement: Supplementary file 1 — Supporting Information [file 41598_2018_35590_MOESM1_ESM.pdf]

Reconstructing the evolutionary history of F_{420}-dependent dehydrogenases

M. Laura Mascotti[1], Hemant Kumar[2], Quoc-Thai Nguyen[2,3,4], Maximiliano Juri Ayub[1], and Marco W. Fraaije[2*]

[1]IMIBIO-SL CONICET, Facultad de Química Bioquímica y Farmacia, Universidad Nacional de San Luis, Ejército de los Andes 950, D5700HHW San Luis, Argentina
[2]Molecular Enzymology Group, University of Groningen, Nijenborgh 4, 9747 AG Groningen, The Netherlands
[3]Scuola Universitaria Superiore IUSS Pavia, Piazza della Vittoria 15, 27100 Pavia, Italy
[4]Faculty of Pharmacy, University of Medicine and Pharmacy, Ho Chi Minh City, 41 Dinh Tien Hoang Street, Ben Nghe Ward, District 1, Ho Chi Minh City, Vietnam

[*]correspondence: Marco W. Fraaije: Molecular Enzymology Group, Groningen Biomolecular Sciences and Biotechnology Institute, University of Groningen, Nijenborgh 4, 9747 AG Groningen, The Netherlands; m.w.fraaije@rug.nl, Tel: +31503634345

Supplementary Information

Fig. S1. Phylogeny of the luciferase-like superfamily

Molecular phylogenetic analysis by Bayesian Inference from full sequences MSA. Posterior probabilities (PP) values are indicated in the nodes. The sequence of protein domain alanine racemase from *Thermaerobacter marianensis* (Uniprot code: E6SIZ8) was used as an external group to root the tree (black branch). The color of the branches indicates: FMN-dependent enzymes (yellow), F_{420}-dependent reductases (purple) and F_{420}-dependent dehydrogenases (green). Taxa names correspond to Uniprot codes. Enzymes previously characterized (biochemically and structurally) are marked with black diamonds and PDB codes (when available) indicated on their names. FSDs characterized in this work are marked with red diamonds.

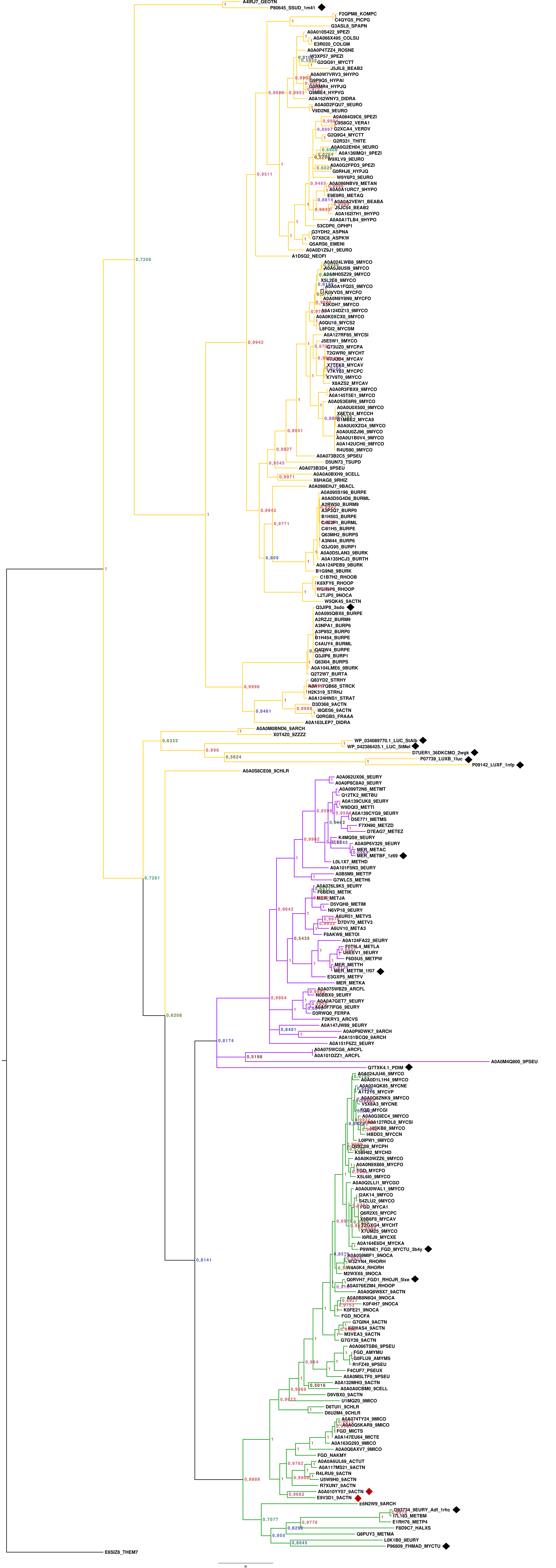

A4IRJ7_GEOTN
P80645_SSUD_1m41
F2QPM8_KOMPC
C4QYG5_PICPG
G3ASL8_SPAPN
A0A010S422_9PEZI
A0A066X495_COLSU
E3R020_COLGM
A0A0P4TZZ4_ROSNE
W3XP57_9PEZI
G2QG91_MYCTT
J5JIL8_BEAB2
A0A0W7VRV3_9HYPO
G9P9Q5_HYPAI
G0RMR4_HYPJQ
G9MIE4_HYPVG
A0A162WNY3_DIDRA
A0A0D2FQU7_9EURO
V9D2N8_9EURO
A0A084G9C6_9PEZI
C3S8G2_VERA1
G2XCA4_VERDV
G2Q9G4_MYCTT
G2R331_THITE
A0A0G2EH04_9EURO
A0A136IMQ1_9PEZI
W9XLV9_9EURO
A0A0G2FPD3_9PEZI
G0RHJ6_HYPJQ
W9Y6P3_9EURO
A0A086NBV9_METAN
A0A0A1URC7_9HYPO
E9E9R5_METAQ
A0A0A2VEW1_BEABA
J5JC54_BEAB2
A0A162I7H1_9HYPO
A0A0A1TLB4_9HYPO
S3CDP0_OPHP1
G3YDH2_ASPNA
G7X8C8_ASPKW
Q5AR56_EMENI
A0A0D1Z9J1_9EURO
A1D5Q2_NEOFI
A0A024LWB8_9MYCO
A0A0U5I9_9MYCO
A0A0H0SZ29_9MYCO
X5L2E8_9MYCO
A0A0A1FQ25_9MYCO
K0VVD5_MYCFO
A0A0N9Y8N9_MYCFO
X5KDH7_9MYCO
A0A124DZ13_9MYCO
A0A0K0XCX0_9MYCO
A0QU18_MYCS2
L8FGI2_MYCSM
A0A127RF85_MYCSI
J5E5W1_9MYCO
G7U3Z0_MYCPA
T2GWR0_MYCHT
A0A0Q5JG94_MYCAV
X7TEK8_MYCAV
V7KY83_MYCPC
X7V9T0_9MYCO
X8AZS2_MYCAV
A0A0R3FBX9_9MYCO
A0A145T5E1_9MYCO
A0A0S3E8R9_9MYCO
A0A0U0X500_9MYCO
X8ETV4_MYCCH
B1MBE2_MYCA9
A0A0U0XZG4_9MYCO
A0A0U0ZJ96_9MYCO
A0A0U1B0V4_9MYCO
A0A142UCH0_9MYCO
R4US90_9MYCO
A0A073B2C5_9PSEU
D5UN73_TSUPD
A0A073B3D4_9PSEU
A0A0A0BXH9_9CELL
X6HAG8_9RHIZ
A0A098EHJ7_9BACL
A0A095S196_BURPE
A0A0D5G4D8_BURML
A2RW50_BURM9
A3P3Q7_BURP0
B1H503_BURPE
C4B2F1_BURML
C4I1H5_BURPE
Q63MH2_BURPS
A3NI44_BURP6
Q3JG95_BURP1
A0A0D5LAN3_9BURK
A0A135HCJ3_BURTH
A0A124PEB9_9BURK
B1G9N8_9BURK
C1B7H2_RHOOB
K8XFY6_RHOOP
V9R9P6_RHOOP
L2TJP5_9NOCA
W5QK45_9ACTN
Q3JIP8_3sdo
A0A095QBX6_BURPE
A2RZJ2_BURM9
A3NPA1_BURP6
A3P9S2_BURP0
B1H454_BURPE
C4AUY4_BURML
C4I3W4_BURPE
Q3JIP8_BURP1
Q63I04_BURPS
A0A104LME6_9BURK
Q2T2W7_BURTA
Q83YD2_STRHY
A0A117QB68_STRCK
H2K319_STRHJ
A0A124HNS1_STRAT
D3D368_9ACTN
I8QES6_9ACTN
Q0RGB5_FRAAA
A0A163LEP7_DIDRA
A0A0M0BND6_9ARCH
X0T4Z0_9ZZZZ
WP_034089770.1_LUC_StAlb
WP_042386425.1_LUC_StMel
D7UER1_36DKCMO_2wgk
P07739_LUXB_1luc
P09142_LUXF_1nfp
A0A0S8CE08_9CHLR
A0A062UX06_9EURY
A0A0P8C8A0_9EURY
A0A099T2N6_METMT
Q12TK2_METBU
A0A139CUK8_9EURY
W9DQI3_METTI
A0A139CYG9_9EURY
D5E771_METMS
F7XN90_METZD
D7EAG7_METEZ
K4MQS9_9EURY
A0A0P6V329_9EURY
MER_METAC
MER_METBF_1z69
L0L1X7_METHD
A0A101F5N3_9EURY
A0B5M9_METTP
G7WLC5_METH6
A0A076L9K5_9EURY
F6BEN3_METIK
MER_METJA
D5VQH8_METIM
N6VP18_9EURY
A6UR51_METVS
D7DV70_METV3
A6UV10_META3
F8AKW8_METOI
A0A124FA22_9EURY
F0T9L4_METLA
U6EEV1_9EURY
F6D5U5_METPW
MER_METTH
MER_METTM_1f07
E3GXP5_METFV
MER_METKA
A0A075WBZ9_ARCFL
N0BBX9_9EURY
A0A0A7GET7_9EURY
A0A0F7IFG6_9EURY
D3RWQ0_FERPA
F2KRY3_ARCVS
A0A147JW89_9EURY
A0A0P9DWK7_9ARCH
A0A151BCQ9_9ARCH
A0A151F6Z2_9EURY
A0A075WCG6_ARCFL
A0A101DZZ1_ARCFL
A0A0M4Q800_9PSEU
Q7TXK4.1_PDIM
A0A024JU46_9MYCO
A0A0D1L1H4_9MYCO
A0A024QK85_MYCNE
A1T2Y6_MYCVP
A0A0Q8ZNK9_9MYCO
V5X8A3_MYCNE
FGD_MYCGI
A0A0G3IEC4_9MYCO
A0A127RDL8_MYCSI
I4SKB8_9MYCO
I4BDD3_MYCCN
L0IPW1_9MYCO
Q2XC09_MYCPH
K5BH82_MYCHD
A0A0K0WZZ6_9MYCO
A0A0N9X869_MYCFO
FGD_MYCFO
X5L6I0_9MYCO
A0A0Q2LLI1_MYCGO
A0A0U0WAL1_9MYCO
I2AK14_9MYCO
S4ZLU2_9MYCO
FGD_MYCA1
Q6R2X5_MYCPC
X8B6F8_MYCAV
T2GXG4_MYCHT
X7UM25_9MYCO
I0REJ9_MYCXE
A0A164E6D4_MYCKA
P9WNE1_FGD_MYCTU_3b4y
A0A059MIF1_9NOCA
W3ZYN4_RHORH
N4A0K4_RHORH
M2WXX6_9NOCA
Q0RVH7_FGD1_RHOJR_5lxe
A0A076EZM4_RHOOP
A0A0Q8W8X7_9ACTN
A0A0B8N8Q4_9NOCA
K0F4H7_9NOCA
K0FE21_9NOCA
FGD_NOCFA
G7GIN4_9ACTN
K6WAS4_9ACTN
M3VEA3_9ACTN
G7GY39_9ACTN
A0A066TSB6_9PSEU
FGD_AMYMU
G0FLU9_AMYMS
R1FZ49_9PSEU
F4CUF7_PSEUX
A0A0M5LTF0_9PSEU
A0A132MHI3_9ACTN
A0A0A0CBM0_9CELL
D9VBX0_9ACTN
U1MQZ0_9MICO
D6TUI1_9CHLR
D6U2M4_9CHLR
A0A074TY24_9MICO
A0A0Q5KAR9_9MICO
FGD_MICTS
A0A147EU64_MICTE
A0A163G293_9MICO
A0A0Q8AXV7_9MICO
FGD_NAKMY
A0A0A6UL69_ACTUT
A0A117MS21_9ACTN
R4LRU9_9ACTN
U5W9H0_9ACTN
R7XUN7_9ACTN
A0A010YY07_9ACTN
E9V3D1_9ACTN
E6N2W9_9ARCH
Q93734_9EURY_Adf_1rhc
I7L183_METBM
E1RH76_METP4
F8D9C7_HALXS
Q8PUY3_METMA
L0K1B0_9EURY
P96809_FMHAD_MYCTU
E6SIZ8_THEM7

Fig. S2. Purification of novel dehydrogenases and ancestral enzymes
SDS-PAGE analysis of purified enzyme fractions: a) FSD-Noca (1) and b) AncD1 (1), FSD-Cryar (2 and 3). Only the marked lanes in the gels are relevant. PageRuler™ Prestained Protein Ladder (10-180 kDa, Thermo Scientific) was used.

a)

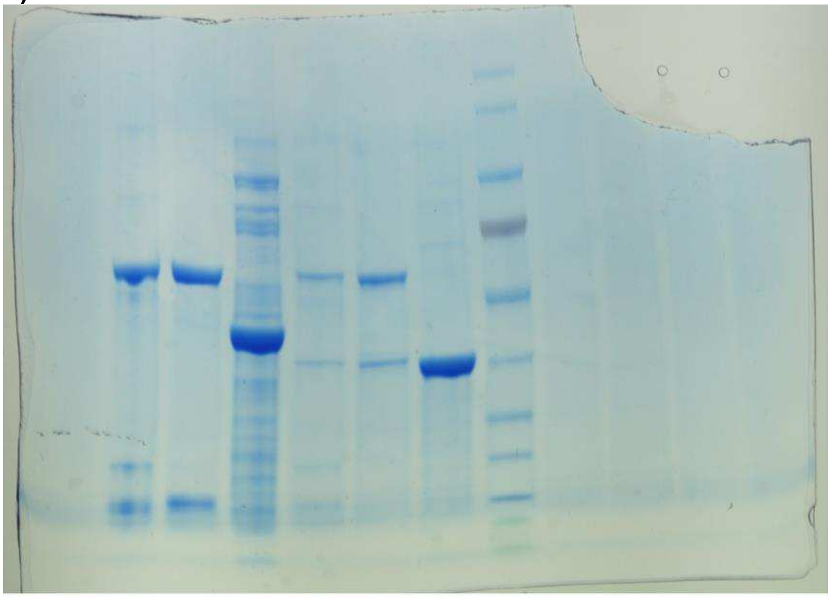

1 M

b)

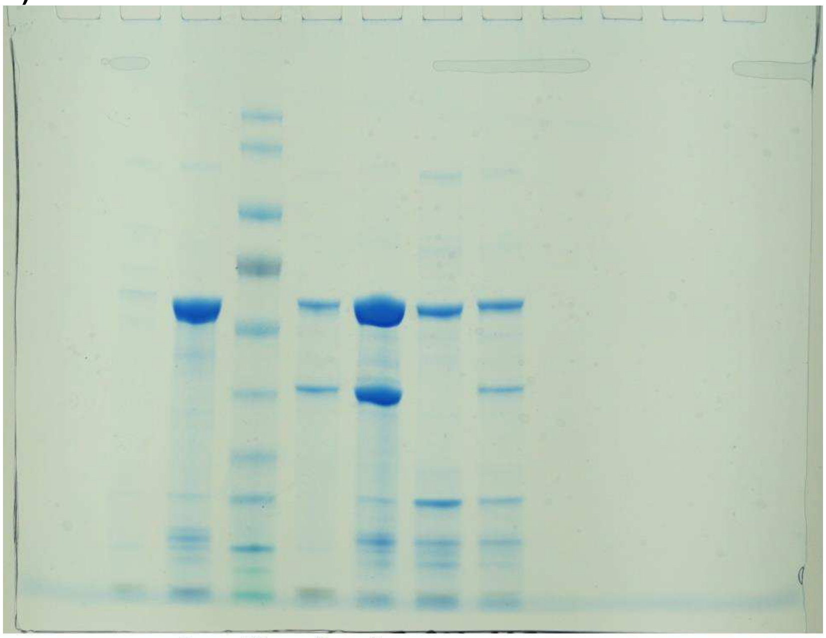

1 M 2 3

Table S1. Colorimetric substrate profile analysis

Substrate	FGD-Noca (FSD-Noca)	FGD-Cryar (FSD-Cryar)	AncD1
D-glucose-6-phosphate	+++	+++	+++
D-fructose-6-phosphate	+++	+++	++
D-mannose-6-phosphate	+++	++	++
D-glucosamine-6-phosphate	+	+	+
D-glucose-1-phosphate	-	-	-
D-galactose-1-phosphate	-	-	-
D-glucose	+	+	+
D-galactose	-	-	-
D-mannose	-	+	-
D-xylose	+	+	+
D-ribose	-	+	-
Isopropanol	-	-	-
Isobutanol	-	-	-
Butanol	-	-	-
Cyclohexanol	-	-	-

Assays were carried out in 96-well plates following the reduction of F_{420} at 400 nm over time. Reaction mixtures (200 µl) contained: 25 µl cell free extracts, 100 µl substrates (50 mM final concentration), 20 µl F_{420} (20 µM final concentration) and 65 µl KPi buffer. Reactions were monitored over first 3 min at regular intervals of 3 s. 10, 15 and 30 min were also recorded. Experiments were repeated two times in duplicate.

Fig. S3. pH optimum of novel dehydrogenases and AncD1

pH dependent relative activities of: a) FSD-Noca, b) AncD1, and c) FSD-Cryar.

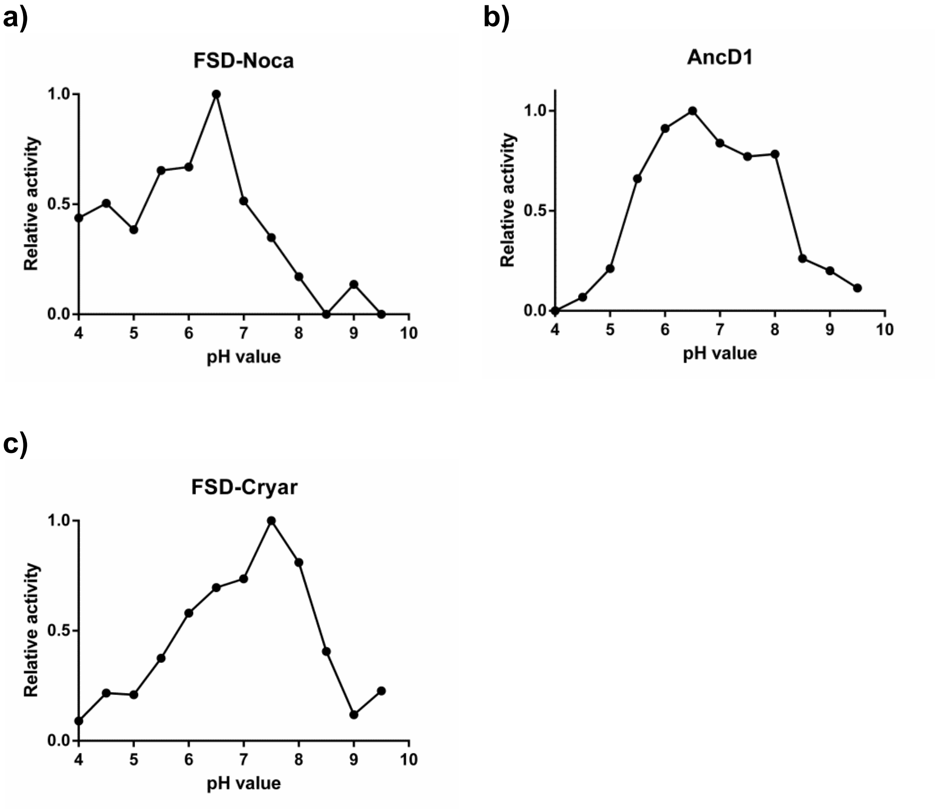

Fig. S4. F_{420} binding assay

Fluorescence spectrum were measured using excitation and emission wavelengths at 420 nm and 475 nm, respectively. Experiments were performed in duplicate. a) FSD-Cryar. b) FSD-Noca.

a) b)

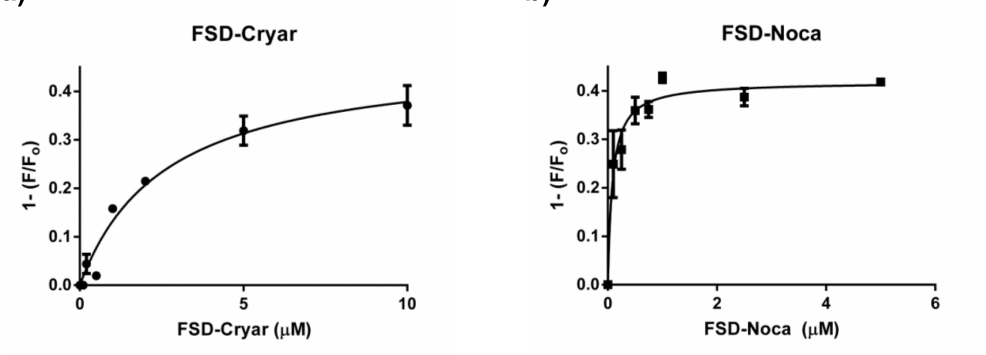

Fig S5. Accuracy of the ancestral reconstructed dehydrogenases

AncDR (overall accuracy of the ancestral sequence = 0.61)

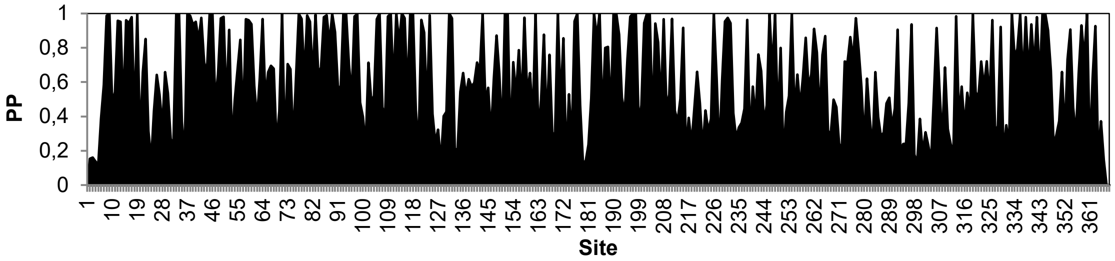

AncD2 (overall accuracy of the ancestral sequence = 0.65)

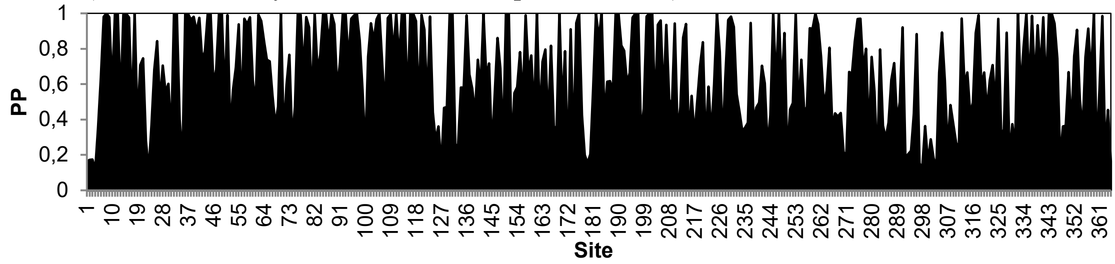

AncD1 (overall accuracy of the ancestral sequence = 0.91)

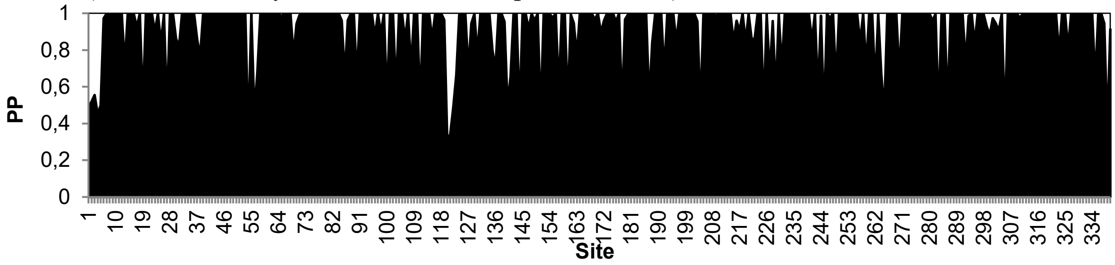

Table S2. Taxonomic distribution of dehydrogenases

Clade	UNIPROT	NAME (tree)	Organism	Taxonomy	Name/PDB
FSDs	A0A010YY07	A0A010YY07_9ACTN	*Cryptosporangium arvum* DSM 44712	Actinobacteria-Frankiales	FSD-Cryar
	E9V3D1	E9V3D1_9ACTN	*Nocardioidaceae* bacterium Broad-1	Actinobacteria-Propionibacteriales	FSD-Noca
	A0A0Q5KAR9	A0A0Q5KAR9_9MICO	*Microbacterium sp.* Leaf151	Actinobacteria-Micrococcales	
	A0A074TY24	A0A074TY24_9MICO	*Microbacterium sp.* Leaf152	Actinobacteria-Micrococcales	
	E8NCH3	FGD_MICTS	*Microbacterium testaceum*	Actinobacteria-Micrococcales	
	A0A147EU64	A0A147EU64_MICTE	*Microbacterium testaceum*	Actinobacteria-Micrococcales	
	A0A163G293	A0A163G293_9MICO	*Microbacterium sp.* T32	Actinobacteria-Micrococcales	
	A0A0Q8AXV7	A0A0Q8AXV7_9MICO	*Microbacterium sp.* Root166	Actinobacteria-Micrococcales	
	C8XBB4	FGD_NAKMY	*Nakamurella multipartite*	Actinobacteria-Nakamurellales	
	A0A0A6UL69	A0A0A6UL69_ACTUT	*Actinoplanes utahensis*	Actinobacteria-Micromonosporales	
	A0A117MS21	A0A117MS21_9ACTN	*Actinoplanes awajinensis* subsp. *mycoplanecinus*	Actinobacteria-Micromonosporales	
	R4LRU9	R4LRU9_9ACTN	*Actinoplanes sp.* N902-109	Actinobacteria-Micromonosporales	
	U5W9H0	U5W9H0_9ACTN	*Actinoplanes friuliensis* DSM 7358	Actinobacteria-Micromonosporales	
	R7XUN7	R7XUN7_9ACTN	*Nocardioides sp.* CF8	Actinobacteria-Propionibacteriales	
	A0A021VMU4	A0A021VMU4_9CELL	*Actinotalea ferrariae* CF5-4	Actinobacteria-Micrococcales	
	A0A0Q6R9Q9	A0A0Q6R9Q9_9MICO	*Yonghaparkia sp.* Root332	Actinobacteria-Micrococcales	
	A0A0Q6ECN2	A0A0Q6ECN2_9MICO	*Microbacterium sp.* Leaf436	Actinobacteria-Micrococcales	
	A0A074TY24	A0A074TY24_9MICO	*Microbacterium sp.* SUBG005	Actinobacteria-Micrococcales	
	A0A0Q8LV00	A0A0Q8LV00_9MICO	*Microbacterium sp.* Root180	Actinobacteria-Micrococcales	
FGDs	A0A024JU46	A0A024JU46_9MYCO	*Mycobacterium triplex*	Actinobacteria-Corynebacteriales	
	A0A0D1L1H4	A0A0D1L1H4_9MYCO	*Mycobacterium llatzerense*	Actinobacteria-Corynebacteriales	
	A0A024QK85	A0A024QK85_MYCNE	*Mycobacterium neoaurum*	Actinobacteria-Corynebacteriales	
	A1T2Y6	A1T2Y6_MYCVP	*Mycobacterium vanbaalenii* DSM 7251	Actinobacteria-Corynebacteriales	
	A0A0Q8ZNK9	A0A0Q8ZNK9_9MYCO	*Mycobacterium sp.* Root265	Actinobacteria-Corynebacteriales	
	V5X8A3	V5X8A3_MYCNE	*Mycobacterium neoaurum* VKM Ac-1815D	Actinobacteria-Corynebacteriales	

	A4T163	FGD_MYCGI	*Mycobacterium gilvum*	Actinobacteria-Corynebacteriales	
	A0A0G3IEC4	A0A0G3IEC4_9MYCO	*Mycobacterium sp.* EPa45	Actinobacteria-Corynebacteriales	
	A0A127RDL8	A0A127RDL8_MYCSI	*Mycobacterium simiae* (*Mycobacterium habana*)	Actinobacteria-Corynebacteriales	
	J4SKB8	J4SKB8_9MYCO	*Mycobacterium colombiense* CECT 3035	Actinobacteria-Corynebacteriales	
	I4BDD3	I4BDD3_MYCCN	*Mycobacterium chubuense* NBB4	Actinobacteria-Corynebacteriales	
	L0IPW1	L0IPW1_9MYCO	*Mycobacterium sp.* JS623	Actinobacteria-Corynebacteriales	
	K5BH82	K5BH82_MYCHD	*Mycobacterium hassiacum* DSM 44199	Actinobacteria-Corynebacteriales	
	Q9XC09	Q9XC09_MYCPH	*Mycobacterium phlei*	Actinobacteria-Corynebacteriales	
	A0A0K0WZZ6	A0A0K0WZZ6_9MYCO	*Mycobacterium goodie*	Actinobacteria-Corynebacteriales	
	A0A0N9X869	A0A0N9X869_MYCFO	*Mycobacterium fortuitum*	Actinobacteria-Corynebacteriales	
	Q9XC11	FGD_MYCFO	*Mycobacterium fortuitum*	Actinobacteria-Corynebacteriales	
	X5L6I0	X5L6I0_9MYCO	*Mycobacterium mageritense* DSM 44476	Actinobacteria-Corynebacteriales	
	A0A0Q2LLI1	A0A0Q2LLI1_MYCGO	*Mycobacterium gordonae*	Actinobacteria-Corynebacteriales	
	A0A0U0WAL1	A0A0U0WAL1_9MYCO	*Mycobacterium bohemicum* DSM 44277	Actinobacteria-Corynebacteriales	
	I2AK14	I2AK14_9MYCO	*Mycobacterium sp.* MOTT36Y	Actinobacteria-Corynebacteriales	
	S4ZLU2	S4ZLU2_9MYCO	*Mycobacterium yongonense* 05-1390	Actinobacteria-Corynebacteriales	
	A0QLV0	FGD_MYCA1	*Mycobacterium avium* 104	Actinobacteria-Corynebacteriales	
	Q6R2X5	Q6R2X5_MYCPC	*Mycobacterium paratuberculosis*	Actinobacteria-Corynebacteriales	
	X8B6F8	X8B6F8_MYCAV	*Mycobacterium avium* subsp. *avium* 2285 (R)	Actinobacteria-Corynebacteriales	
	T2GXG4	T2GXG4_MYCHT	*Mycobacterium avium* subsp. *Hominissuis* TH135	Actinobacteria-Corynebacteriales	
	X7UM25	X7UM25_9MYCO	*Mycobacterium sp.* MAC_080597_8934	Actinobacteria-Corynebacteriales	
	I0REJ9	I0REJ9_MYCXE	*Mycobacterium xenopi* RIVM700367	Actinobacteria-Corynebacteriales	
	A0A164E6D4	A0A164E6D4_MYCKA	*Mycobacterium kansasii*	Actinobacteria-Corynebacteriales	
	P9WNE1	FGD_MYCTU	*Mycobacterium tuberculosis* H37Rv	Actinobacteria-Corynebacteriales	3B4Y
	A0A059MIF1	A0A059MIF1_9NOCA	*Rhodococcus aetherivorans*	Actinobacteria-Corynebacteriales	
	W3ZYN4	W3ZYN4_RHORH	*Rhodococcus rhodochrous* ATCC 21198	Actinobacteria-Corynebacteriales	
	W4A0K4	W4A0K4_RHORH	*Rhodococcus rhodochrous* ATCC 21198	Actinobacteria-Corynebacteriales	
	M2WXX6	M2WXX6_9NOCA	*Rhodococcus triatomae* BKS 15-14	Actinobacteria-Corynebacteriales	

	A0A076EZM4	A0A076EZM4_RHOOP	*Rhodococcus opacus* (*Nocardia opaca*)	Actinobacteria-Corynebacteriales	
	Q0RVH7	FGD1_RHOJR	*Rhodococcus jostii RHA1*	Actinobacteria-Corynebacteriales	5LXE
	A0A0Q8W8X7	A0A0Q8W8X7_9ACTN	*Aeromicrobium sp.* Root236	Actinobacteria-Propionibacteriales	
	A0A0B8N8Q4	A0A0B8N8Q4_9NOCA	*Nocardia seriolae*	Actinobacteria-Corynebacteriales	
	K0F4H7	K0F4H7_9NOCA	*Nocardia brasiliensis* ATCC 700358	Actinobacteria-Corynebacteriales	
	K0FE21	K0FE21_9NOCA	*Nocardia brasiliensis* ATCC 700358	Actinobacteria-Corynebacteriales	
	Q5YNN4	FGD_NOCFA	*Nocardia farcinica* IFM 10152	Actinobacteria-Corynebacteriales	
	G7GIN4	G7GIN4_9ACTN	*Gordonia amarae* NBRC 15530	Actinobacteria-Corynebacteriales	
	K6WAS4	K6WAS4_9ACTN	*Gordonia rhizosphera* NBRC 16068	Actinobacteria-Corynebacteriales	
	M3VEA3	M3VEA3_9ACTN	*Gordonia paraffinivorans* NBRC 108238	Actinobacteria-Corynebacteriales	
	G7GY39	G7GY39_9ACTN	*Gordonia araii* NBRC 100433	Actinobacteria-Corynebacteriales	
	A0A066TSB6	A0A066TSB6_9PSEU	*Amycolatopsis rifamycinica*	Actinobacteria-Pseudonocardiales	
	D8I5S8	FGD_AMYMU	*Amycolatopsis mediterranei* U-32	Actinobacteria-Pseudonocardiales	
	G0FLU9	G0FLU9_AMYMS	*Amycolatopsis mediterranei* S699 *(Nocardia mediterranei)*	Actinobacteria-Pseudonocardiales	
	R1FZ49	R1FZ49_9PSEU	*Amycolatopsis vancoresmycina* DSM 44592	Actinobacteria-Pseudonocardiales	
	F4CUF7	F4CUF7_PSEUX	*Pseudonocardia dioxanivorans*	Actinobacteria-Pseudonocardiales	
	A0A0M5LTF0	A0A0M5LTF0_9PSEU	*Pseudonocardia sp.* HH130629-09	Actinobacteria-Pseudonocardiales	
	A0A132MHI3	A0A132MHI3_9ACTN	*Streptomyces thermoautotrophicus*	Actinobacteria-Streptomycetales	
	A0A0A0CBM0	A0A0A0CBM0_9CELL	*Actinotalea fermentans* ATCC 43279	Actinobacteria- Micrococcales	
	D9VBX0	D9VBX0_9ACTN	*Streptomyces sp.* AA4	Actinobacteria-Streptomycetales	
	U1MQZ0	U1MQZ0_9MICO	*Agrococcus pavilionensis* RW1	Actinobacteria- Micrococcales	
	D6TUI1	D6TUI1_9CHLR	*Ktedonobacter racemifer* DSM 44963	Chloroflexi- Ktedonobacteria- Ktedonobacterales	
	D6U2M4	D6U2M4_9CHLR	*Ktedonobacter racemifer* DSM 44963	Chloroflexi- Ktedonobacteria- Ktedonobacterales	
**ADHs**	P96809	P96809_FHMAD_MYCTU	*Mycobacterium tuberculosis* H37Rv	Actinobacteria-Corynebacteriales	FHMAD_Myctu
	F8D9C7	F8D9C7_HALXS	*Halopiger xanaduensis* DSM 18323	Euryarchaeota-Halobacteria-Natrialbales	
	E6N2W9	E6N2W9_9ARCH	*Candidatus Caldiarchaeum subterraneum*	Thaumarchaeota-unclassified Thaumarchaeota	
	O93734	O93734_Adf_1rhc	*Methanoculleus thermophiles*	Euryarchaeota-Methanomicrobia-Methanomicrobiales	1RHC
	L0K1B0	L0K1B0_9EURY	*Natronococcus occultus* SP4	Euryarchaeota-Halobacteria-Natrialbales	

	Q8PUY3	Q8PUY3_METMA	*Methanosarcina mazei* ATCC BAA-159	Euryarchaeota-Methanomicrobia-Methanosarcinales	
	E1RH76	E1RH76_METP4	*Methanolacinia petrolearia* DSM 11571	Euryarchaeota-Methanomicrobia-Methanomicrobiales	
	I7L183	I7L183_METBM	*Methanoculleus bourgensis* ATCC 43281	Euryarchaeota-Methanomicrobia-Methanomicrobiales	

Fig. S6. Estimated emergence time of sugar dehydrogenase activity

Tree displaying FGDs and FSDs subfamilies classified by taxonomic distribution as follows: Chloroflexi–Ktedonobacteria–Ktedonobacterales (black circles), Actinobacteria–Micrococcales (red circles), Actinobacteria–Streptomycetales (blue circles), Actinobacteria–Pseudonocardiales (orange circles), Actinobacteria–Propionibacteriales (pink circles), Actinobacteria–Frankiales (cyan circles), Actinobacteria–Corynebacteriales (grey circles), Actinobacteria–Micromonosporales (yellow circles) and Actinobacteria–Nakamurellales (lilac circles). Divergence time image was obtained using the TimeTree web server[1]. Emergence of sugar dehydrogenase functionality dates at least 3069 mya according to the taxonomic distribution observed in the tree.

[1] http://www.timetree.org/. Hedges SB, Marin J, Suleski M, Paymer M, Kumar S. Tree of Life Reveals Clock-Like Speciation and Diversification. *Mol. Biol. Evol.* **2015**. 32(4):835–845 doi:10.1093/molbev/msv037

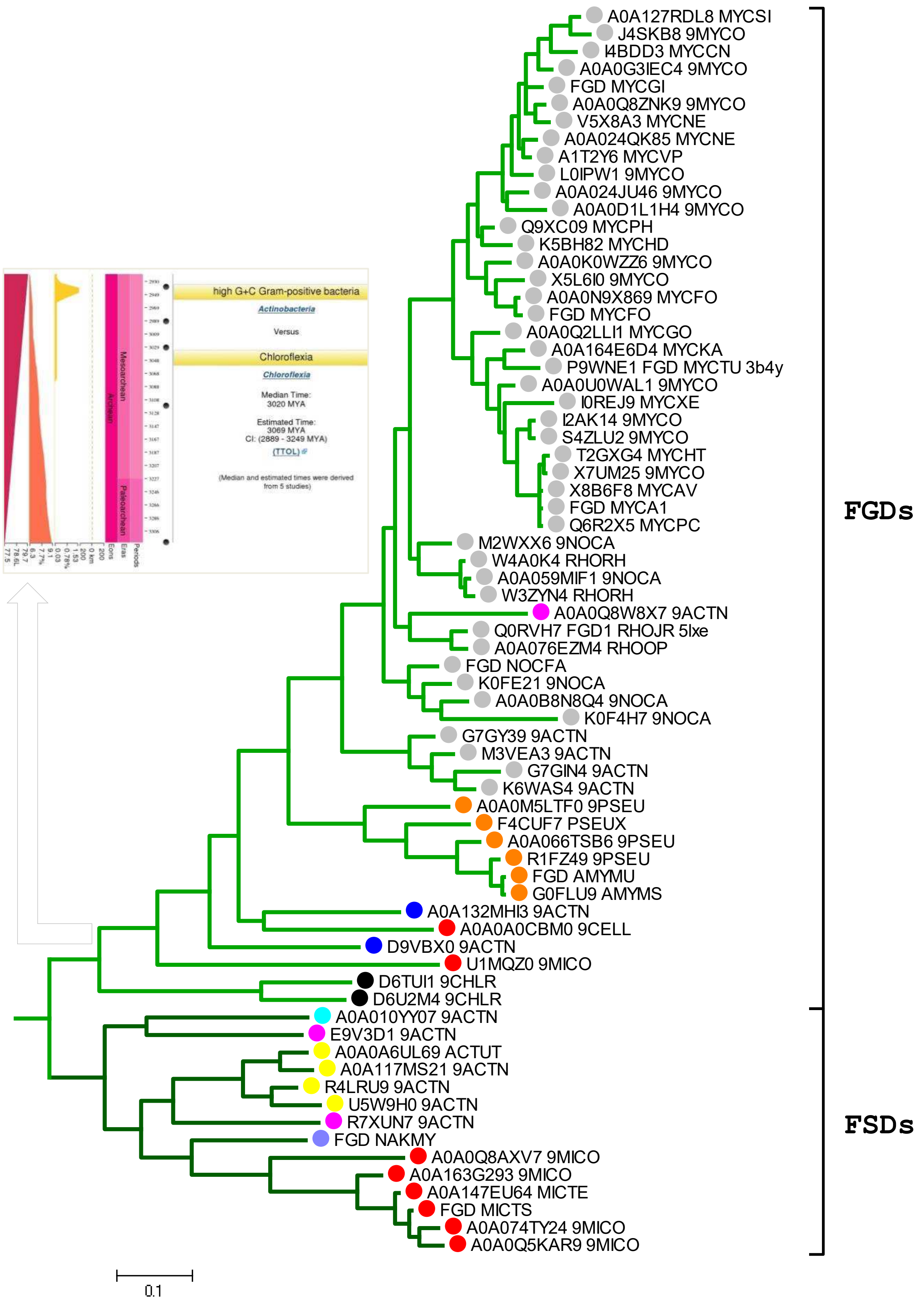
A0A127RDL8 MYCSI
J4SKB8 9MYCO
I4BDD3 MYCCN
A0A0G3IEC4 9MYCO
FGD MYCGI
A0A0Q8ZNK9 9MYCO
V5X8A3 MYCNE
A0A024QK85 MYCNE
A1T2Y6 MYCVP
L0IPW1 9MYCO
A0A024JU46 9MYCO
A0A0D1L1H4 9MYCO
Q9XC09 MYCPH
K5BH82 MYCHD
A0A0K0WZZ6 9MYCO
X5L6I0 9MYCO
A0A0N9X869 MYCFO
FGD MYCFO
A0A0Q2LLI1 MYCGO
A0A164E6D4 MYCKA
P9WNE1 FGD MYCTU 3b4y
A0A0U0WAL1 9MYCO
I0REJ9 MYCXE
I2AK14 9MYCO
S4ZLU2 9MYCO
T2GXG4 MYCHT
X7UM25 9MYCO
X8B6F8 MYCAV
FGD MYCA1
Q6R2X5 MYCPC
M2WXX6 9NOCA
W4A0K4 RHORH
A0A059MIF1 9NOCA
W3ZYN4 RHORH
A0A0Q8W8X7 9ACTN
Q0RVH7 FGD1 RHOJR 5lxe
A0A076EZM4 RHOOP
FGD NOCFA
K0FE21 9NOCA
A0A0B8N8Q4 9NOCA
K0F4H7 9NOCA
G7GY39 9ACTN
M3VEA3 9ACTN
G7GIN4 9ACTN
K6WAS4 9ACTN
A0A0M5LTF0 9PSEU
F4CUF7 PSEUX
A0A066TSB6 9PSEU
R1FZ49 9PSEU
FGD AMYMU
G0FLU9 AMYMS
A0A132MHI3 9ACTN
A0A0A0CBM0 9CELL
D9VBX0 9ACTN
U1MQZ0 9MICO
D6TUI1 9CHLR
D6U2M4 9CHLR
A0A010YY07 9ACTN
E9V3D1 9ACTN
A0A0A6UL69 ACTUT
A0A117MS21 9ACTN
R4LRU9 9ACTN
U5W9H0 9ACTN
R7XUN7 9ACTN
FGD NAKMY
A0A0Q8AXV7 9MICO
A0A163G293 9MICO
A0A147EU64 MICTE
FGD MICTS
A0A074TY24 9MICO
A0A0Q5KAR9 9MICO
FGDs
FSDs
0.1
high G+C Gram-positive bacteria
Actinobacteria
Versus
Chloroflexia
Chloroflexia
Median Time:
3020 MYA
Estimated Time:
3069 MYA
CI: (2889 - 3249 MYA)
(TTOL)
(Median and estimated times were derived from 5 studies)
Mesoarchean
Archean
Paleoarchean
Eons
Eras
Periods

Fig. S7. Structural analysis of substrate cavity

a) From left to right, surfaces of: Adf from *Methanoculleus thermophilus* (PDB 1RHC, pink color), FGD-Myctu from *Mycobacterium tuberculosis* (PDB 3B4Y, purple color) and AncD1 (green color). F_{420} cofactor is shown in yellow sticks. For AncD1 model, F_{420} cofactor corresponds to the overlapping with 3B4Y structure. b) Left: Substrate cavity residues from 1RHC (in pink) overlapped with AncD1 (in green). Right: Substrate cavity residues from 3B4Y (in purple) overlapped with AncD1 (in green). F_{420} cofactor is shown in yellow sticks. Met175 in 1RHC was subjected to evolutionary trajectory analysis showing the specific switch to Ser177 in AncD1. From this ancestor, FSDs and FGDs emerged. While FSDs retained the Ser in that position, in FGDs it changed to Gly (175 in 3B4Y). Other residues defining the substrate pocket (involved in the interaction with the phosphate moiety of G6P in FGDs) are indicated as reference.

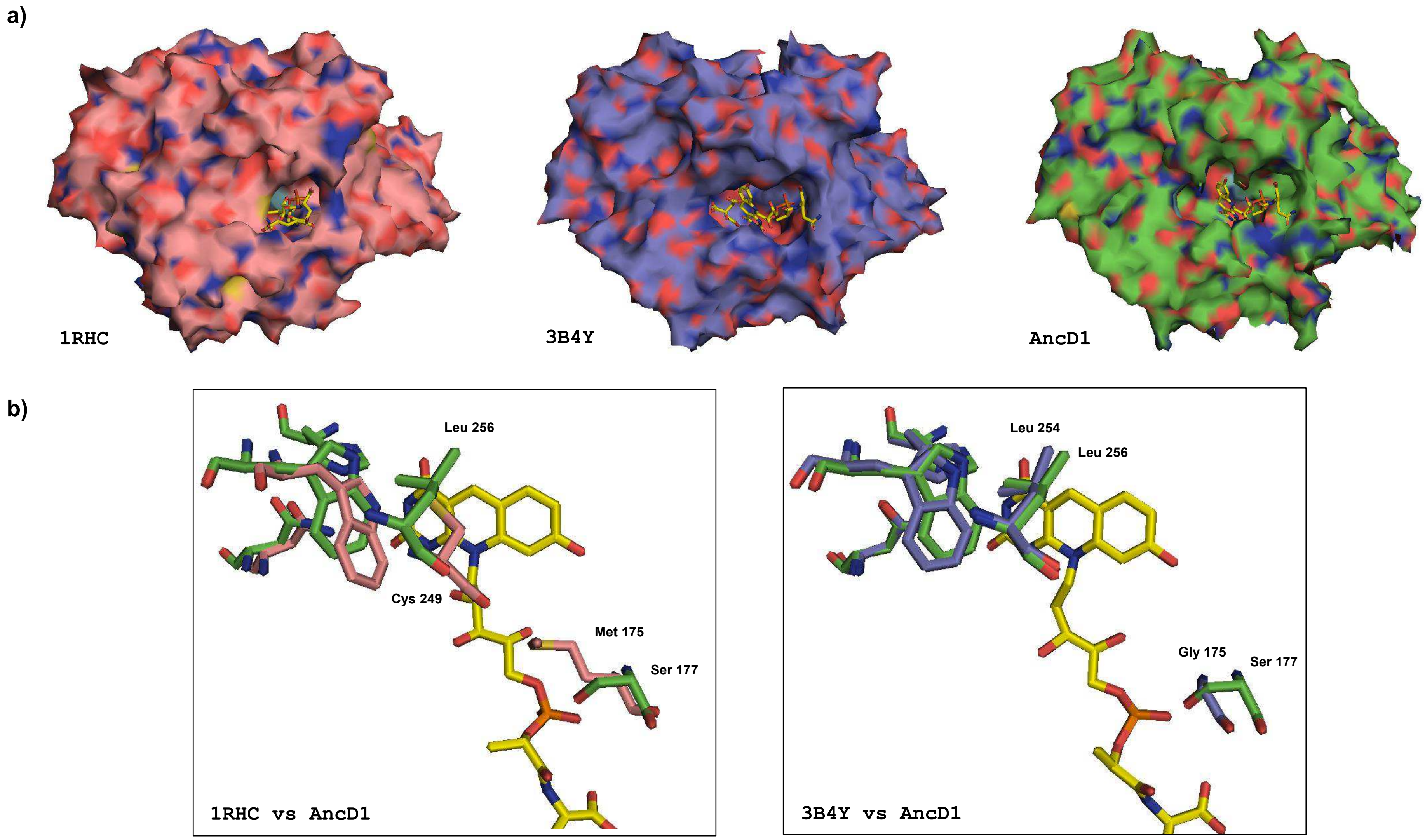
a)
1RHC
3B4Y
AncD1
b)
Leu 256
Cys 249
Met 175
Ser 177
1RHC vs AncD1
Leu 254
Leu 256
Gly 175
Ser 177
3B4Y vs AncD1

Data S1. Fig S1 tree (newick format)

((((A4IRJ7_GEOTN:0.13702229999999993,P80645_SSUD_1m41:0.3378608999999999):0.64
5721000000002,((((((F2QPM8_KOMPC:0.03353621999999978,C4QYG5_PICPG:0.002821050
0000001026):0.31351629999999986,G3ASL8_SPAPN:0.2885783000000002):0.16873890000
000014,(((((A0A010S422_9PEZI:0.01659963999999916,(A0A066X495_COLSU:0.02275679
99999998,E3R020_COLGM:0.02739678000000012):0.03941939999999988):0.048785389999
99993,A0A0P4TZZ4_ROSNE:0.06689456000000016,(W3XP57_9PEZI:0.06476251999999993,(
G2QG91_MYCTT:0.05117381999999814,J5JIL8_BEAB2:0.1481314000000002):0.06574658
999999983):0.022840470000000224,((A0A0W7VRV3_9HYPO:0.007961085999999895,G9P9Q5
_HYPAI:0.001974254999999925):0.0310839899999995,(G0RMR4_HYPJQ:0.0140206100000
00211,G9MIE4_HYPVG:0.01195252999999985):0.016449090000000055):0.04995779000000
011):0.02148210000000006,A0A162WNY3_DIDRA:0.09838468000000011):0.0459237599999
9998,(A0A0D2FQU7_9EURO:0.026654119999999892,V9D2N8_9EURO:0.009124427000000157)
:0.1599360999999991):0.058777220000000074,((((((A0A084G9C6_9PEZI:0.074395389
99999984,(C9S8G2_VERA1:0.007755600999999945,G2XCA4_VERDV:0.0024125100000009):
0.0794397400000002):0.0403177299999994,(G2Q9G4_MYCTT:0.02811400999999991,G2R3
31_THITE:0.06985556000000015):0.0462974799999978):0.02076190000000011,((A0A0G
2EH04_9EURO:0.0646334799999991,A0A136IMQ1_9PEZI:0.1214016999999983):0.029509
3500000001,W9XLV9_9EURO:0.07098174000000013):0.02614510999999986,(A0A0G2FPD3_9
PEZI:0.09868799000000017,G0RHJ6_HYPJQ:0.11582030000000021):0.01661958999999990
6,W9Y6P3_9EURO:0.1644866999999987):0.028691760000000066,((((A0A086NBV9_METAN:
0.00771061699999864,A0A0A1URC7_9HYPO:0.008247976999999906):0.0168208000000000
8,E9E9R5_METAQ:0.011508730000000078):0.059712160000000125,((A0A0A2VEW1_BEABA:0
.0023348450000003,J5JC54_BEAB2:0.007839054999999817):0.028828920000000036,A0A
162I7H1_9HYPO:0.03871637999999884):0.08911883999999981):0.0202918699999988,A
0A0A1TLB4_9HYPO:0.1077376000000001):0.03194338999999905):0.02633875999999795
,S3CDP0_OPHP1:0.07305887000000011):0.0802192299999997,((G3YDH2_ASPNA:0.007628
264000000051,G7X8C8_ASPKW:0.011951359999999855):0.04736564000000021,Q5ARS6_EME
NI:0.03796097999999981):0.059679570000000126):0.0447409599999994,A0A0D1Z9J1_9
EURO:0.1182365999999991):0.06501833999999995):0.09077034000000017):0.08391631
000000022,A1D5Q2_NEOFI:0.25413010000000025):0.0656647000000013,(((((((((((((A
0A024LWB8_9MYCO:0.00542900999999845,A0A0J8U5I9_9MYCO:0.00521094299999801):0.
00435730199999841,A0A0H0SZ29_9MYCO:0.00380477099999846):0.02640388000000015
7,X5L2E8_9MYCO:0.0127326399999985):0.00814013299999938,((A0A0A1FQ25_9MYCO:0.
0020689040000000149,K0VVD5_MYCFO:0.002182835999997993):0.01778316000000002,A0A
0N9Y8N9_MYCFO:0.005410679999999779):0.03209373000000154):0.0088647169999998,X
5KDH7_9MYCO:0.04173043999999981):0.006797134999999788,A0A124DZ13_9MYCO:0.03889
60100000002):0.0353340000000002,(A0A0K0XCX0_9MYCO:0.003761878000000163,(A0QU18
_MYCS2:0.005149965999999839,L8FGI2_MYCSM:0.008413228000000217):0.019405069999
99913):0.0333527799999997):0.025535389999999936,((A0A127RF85_MYCSI:0.06601298
,(J5E5W1_9MYCO:0.02390932999999995,(((Q73UZ0_MYCPA:0.0021984179999998688,T2GWR
0_MYCHT:0.002051255000000296,V7JQS4_MYCAV:0.0022808219999999046,X7TEK8_MYCAV:
0.00556979600000071,V7KY83_MYCPC:0.005001422999999949):0.005228502000000024,X
7V9T0_9MYCO:0.005076870999999983):0.005367552000000053,X8AZS2_MYCAV:0.05090101
0000000024):0.05174504000000013):0.023427690000000112):0.021404689999999782,((
A0A0R3FBX9_9MYCO:0.005047315000000108,A0A145T5E1_9MYCO:0.001965218000000463):
0.0632524100000004,(A0A0S3E8R9_9MYCO:0.02485916000000102,((A0A0U0X500_9MYCO:
0.0019693539999998677,X8ETV4_MYCCH:0.002385626000001406,B1MBE2_MYCA9:0.002234
010000000085,A0A0U0XZG4_9MYCO:0.005610915000000105):0.005441868000000127,A0A0
U0ZJ96_9MYCO:0.002130847000001023,A0A0U1B0V4_9MYCO:0.0022640649999998708,A0A1
42UCH0_9MYCO:0.002066775999999937,R4US90_9MYCO:0.004997202999999839):0.0831949
8999999997):0.0344579700000006):0.05953321000000114):0.03062484000000021):0.
0695128500000018,A0A073B2C5_9PSEU:0.1346913999999999):0.07691727999999998,D5U
N73_TSUPD:0.27731379999999994):0.0808019199999986,A0A073B3D4_9PSEU:0.16424389
999999978):0.05443581000000195,(A0A0A0BXH9_9CELL:0.2478715999999986,X6HAG8_9
RHIZ:0.2495881999999982):0.0757499099999986):0.0610360199999997,((A0A098EHJ
7_9BACL:0.2532573,((((A0A095S196_BURPE:0.002191224000000158,(A0A0D5G4D8_BURML:
0.002387048000001137,A2RW50_BURM9:0.00232424899999945,A3P3Q7_BURP0:0.0020181
589999999083,B1H503_BURPE:0.0021611049999998855,C4B2F1_BURML:0.00222880000000
1418,C4I1H5_BURPE:0.002373901999999833,Q63MH2_BURPS:0.002306425000000001,A3NI
44_BURP6:0.0022537850000001747,Q3JG95_BURP1:0.0022031509999997922):0.005155331
000000096):0.01320237000000013,A0A0D5LAN3_9BURK:0.013042029999999816,A0A135HCJ
3_BURTH:0.01994010999999844):0.026129360000001,A0A124PEB9_9BURK:0.0087637100
00000202):0.05688066000000003,B1G9N8_9BURK:0.06151808999999986):0.243293399999
99977):0.06065319000000023,((C1B7H2_RHOOB:0.04119966000000019,(K8XFY6_RHOOP:0
.0022094269999999305,W8H9P6_RHOOP:0.002121800999999232,L2TJP5_9NOCA:0.0052337
7500000218):0.0159825399999999):0.0756824100000002,W5QK45_9ACTN:0.150425999

9999995):0.2806307000000001):0.03664349999999983):0.0921566600000011):0.30043
42000000002,((((Q3JIP8_3sdo:0.008840492000000033,A0A095QBX6_BURPE:0.0022414419
999998714,A2RZJ2_BURM9:0.0021101300000001544,A3NPA1_BURP6:0.002151116999999924
7,A3P9S2_BURP0:0.002154630999999796,B1H454_BURPE:0.0023030170000000183,C4AUY4_
BURML:0.002088274000000112,C4I3W4_BURPE:0.002114151999998692,Q3JIP8_BURP1:0.0
01981655999998873,Q63I04_BURPS:0.0018629030000001379):0.0372270800000008,A0A
104LME6_9BURK:0.011457430000000102):0.00817956499999833,Q2T2W7_BURTA:0.016966
710000000162):0.4703900999999999,((((Q83YD2_STRHY:0.019582660000000196,A0A117Q
B68_STRCK:0.0024951510000001953,H2K319_STRHJ:0.001988057000000154):0.017311279
99999993,A0A124HNS1_STRAT:0.021004340000000177):0.1565761999999994,(D3D368_9A
CTN:0.1123493999999982,(I8QES6_9ACTN:0.0322284000000016,Q0RGB5_FRAAA:0.02077
434000000002):0.1208317999999993):0.0924472600000014):0.2067527,A0A163LEP7_
DIDRA:0.360905900000001):0.0860086299999981):0.2711939999999994):0.52138940
00000001):0.2266740000000026,(((A0A0M0BND6_9ARCH:0.1234275000000002,X0T4Z0_9
ZZZZ:0.2485595999999988):0.5633932999999998,(('WP_034089770.1_LUC_StAlb':0.08
27534100000003,'WP_042386425.1_LUC_StMel':0.0299332900000000223):1.003972,(D7U
ER1_36DKCMO_2wgk:1.364008,(P07739_LUXB_1luc:0.3910817999999998,P09142_LUXF_1nf
p:0.7710661999999999):1.031367):0.1456235999999996):0.3186679999999973):0.13
06031000000008,(A0A0S8CE08_9CHLR:0.5576469000000002,((((((((A0A062UX06_9EURY:
0.0399383300000002,A0A0P8C8A0_9EURY:0.0332861399999999964):0.1027413999999998
,((((A0A099T2N6_METMT:0.0109899899999999894,Q12TK2_METBU:0.02786343000000022):
0.0258770600000007,((A0A139CUK8_9EURY:0.024226370000000053,W9DQI3_METTI:0.01
594944999999992):0.0197930699999968,((A0A139CYG9_9EURY:0.0104070199999999934,
D5E771_METMS:0.00635021299999938):0.0548672900000001,(F7XN90_METZD:0.0703758
5000000002,D7EAG7_METEZ:0.1319429999999992):0.045997990000000044):0.051492670
00000002):0.011467660000000102):0.0333874999999999,(K4MQS9_9EURY:0.04549059999
999994,(A0A0P6V329_9EURY:0.021695100000000078,(MER_METAC:0.02981338999999994,M
ER_METBF_1z69:0.02899056999999938):0.00986981399999977):0.14113390000000003)
:0.02010914999999991):0.0235416699999996,L0L1X7_METHD:0.0468302599999999):0.0
7085892000000005):0.0919214699999995,A0A101F5N3_9EURY:0.1908649):0.0610170100
0000004,(A0B5M9_METTP:0.1646973000000005,G7WLC5_METH6:0.1493123999999999):0.1
257546999999989):0.1302794999999988,(((((A0A076L9K5_9EURY:0.0103858400000000
9,F6BEN3_METIK:0.01809418000000007):0.0111417799999999,MER_METJA:0.0242307100
000001,(D5VQH8_METIM:0.0580754199999999,N6VP18_9EURY:0.0413097099999999944):0.
0648979499999998):0.049089630000000106,(((A6UR51_METVS:0.08511636,D7DV70_METV
3:0.10067500000000007):0.03355511,A6UV10_META3:0.08265081000000007):0.03146756
000000006,F8AKW8_METOI:0.0627719099999993):0.0607590899999999905):0.0922795300
0000033,((A0A124FA22_9EURY:0.1656116999999992,(((F0T9L4_METLA:0.0343776600000
0009,U6EEV1_9EURY:0.01922950000000001):0.0143581100000009,F6D5U5_METPW:0.0526
8672000000076):0.0524150999999994,(MER_METTH:0.0121036999999994,MER_METTM_1
f07:0.0080356399999999):0.0117024900000001):0.0866113399999993):0.0460540300
0000008,E3GXP5_METFV:0.1025183000000006):0.1398455000000003):0.03837591000000
007,MER_METKA:0.3464194000000027):0.0802237799999986):0.0503998699999968,((
(A0A075WBZ9_ARCFL:0.0568750300000005,N0BBX9_9EURY:0.045600319999999916):0.036
53795000000004,((A0A0A7GET7_9EURY:0.0394060599999991,A0A0F7IFG6_9EURY:0.02291
533999999995):0.029258020000000107,D3RWQ0_FERPA:0.034585620000000095):0.022844
78000000001):0.0784090299999999,F2KRY3_ARCVS:0.2050784999999994):0.166556399
99999972,(A0A147JW89_9EURY:0.2922206000000005,(A0A0P9DWK7_9ARCH:0.31385219999
99999,A0A151BCQ9_9ARCH:0.2816982999999974):0.1402271000000016):0.07201749999
999985,A0A151F6Z2_9EURY:0.4253733999999998):0.3862424000000043,((A0A075WCG6_A
RCFL:0.0023855470000000434,A0A101DZZ1_ARCFL:0.0022183000000000064):0.490286600
00000013,A0A0M4Q800_9PSEU:1.786762):0.2093923000000002,'Q7TXK4.1_PDIM':1.09425
30000000001):0.1607101999999958,((E6N2W9_9ARCH:0.7067012000000004,(((((O93734
_9EURY_Adf_1rhc:0.0172825099999999807,I7L183_METBM:0.012063340000000089):0.0759
622200000008,E1RH76_METP4:0.0832575900000001):0.5789255999999998,F8D9C7_HALXS
:0.6859645999999997):0.1055094000000031,Q8PUY3_METMA:0.5055320000000001):0.11
18461999999956,(L0K1B0_9EURY:0.6751613999999999,P96809_FHMAD_MYCTU:0.69580519
99999997):0.1431814999999985):0.0701506400000049):0.1338879999999978,((((((
((((((((A0A024JU46_9MYCO:0.026827429999999985,A0A0D1L1H4_9MYCO:0.050384000000
000206):0.0058722129999999595,(((A0A024QK85_MYCNE:0.0169465699999999994,A1T2Y6_
MYCVP:0.008983935999999915):0.005653118999999762,((A0A0Q8ZNK9_9MYCO:0.01208147
9999999978,V5X8A3_MYCNE:0.01784814999999984):0.012522900000000003,FGD_MYCGI:0.
0208010600000015,(A0A0G3IEC4_9MYCO:0.017485250000000008,((A0A127RDL8_MYCSI:0.
011790650000000014,J4SKB8_9MYCO:0.025571330000000003):0.026114320000000024,I4B
DD3_MYCCN:0.0332392099999963):0.016735940000000227):0.0166155799999999852):0.0
09450715999999915):0.009530468999999986,L0IPW1_9MYCO:0.0263892499999981):0.01
184001000000234):0.0331106199999998,(Q9XC09_MYCPH:0.0144584499999979,K5BH82
_MYCHD:0.0380257299999976):0.005933201000000388):0.01281433999999813,(A0A0K0

WZZ6_9MYCO:0.03142705000000001,((A0A0N9X869_MYCFO:0.003931974999999765,FGD_MY
CFO:0.006975796999999995):0.025871210000000033,X5L6I0_9MYCO:0.0358067800000015
):0.0130530499999999):0.02396632999999925):0.019792240000000128,(A0A0Q2LLI1_M
YCGO:0.03440003999999965,((A0A0U0WAL1_9MYCO:0.018884460000000214,(((I2AK14_9M
YCO:0.005188927999999926,S4ZLU2_9MYCO:0.005469871999999931):0.016504019999997
3,(FGD_MYCA1:0.0022296879999998964,Q6R2X5_MYCPC:0.002356644000000241,X8B6F8_MY
CAV:0.002180585000000068,(T2GXG4_MYCHT:0.005268454999999995,X7UM25_9MYCO:0.0023
659440000001197):0.005680974000000116):0.02933675999999963):0.0165160600000002
77,I0REJ9_MYCXE:0.06298290000000017):0.007008579999999931):0.015896080000000009
,(A0A164E6D4_MYCKA:0.02519676000000004,P9WNE1_FGD_MYCTU_3b4y:0.045172860000000
09):0.022648690000000027):0.01974317999999986):0.025491979999999997):0.05701613
0000000054,(((A0A059MIF1_9NOCA:0.005511923000000252,W3ZYN4_RHORH:0.01070429000
000006):0.006316988999999662,W4A0K4_RHORH:0.004198205999999871):0.055424980000
00018,M2WXX6_9NOCA:0.04129920000000009):0.011518310000000032):0.01983113000000
003,((Q0RVH7_FGD1_RHOJR_5lxe:0.01928579000000008,A0A076EZM4_RHOOP:0.0198213799
99999778):0.05511900999999986,A0A0Q8W8X7_9ACTN:0.1535791999999998):0.019504910
00000021):0.015172529999999629,(((A0A0B8N8Q4_9NOCA:0.03451143000000023,K0F4H7_
9NOCA:0.15191890000000008):0.023442689999999544,K0FE21_9NOCA:0.035193389999999
88):0.019026150000000186,FGD_NOCFA:0.03757639999999984):0.03328945999999977):0
.05489345000000023,(((G7GIN4_9ACTN:0.05791527000000074,K6WAS4_9ACTN:0.0256231
49999999928):0.030601320000000154,M3VEA3_9ACTN:0.0281652199999999):0.02959646
0000000047,G7GY39_9ACTN:0.03188440999999974):0.0891072099999988):0.071113179
99999978,(((A0A066TSB6_9PSEU:0.02968377000000011,((FGD_AMYMU:0.00206522299999
783,G0FLU9_AMYMS:0.002030490000000107):0.01528116999999733,R1FZ49_9PSEU:0.010
29304999999582):0.04481794000000017):0.06740864000000002,F4CUF7_PSEUX:0.08322
556000000025):0.05494403000000144,A0A0M5LTF0_9PSEU:0.11081360000000018):0.101
05249999999977):0.0660713300000002,(A0A132MHI3_9ACTN:0.17894070000000006,A0A0A
0CBM0_9CELL:0.2223647999999998):0.03357048000000029):0.03827943999999972,D9VBX
0_9ACTN:0.1972909999999988):0.06899519000000032,U1MQZ0_9MICO:0.37142770000000
036):0.07691280000000011,(D6TUI1_9CHLR:0.11780599999999986,D6U2M4_9CHLR:0.1104
270000000005):0.2143961000000001):0.06625762999999996,(((((((A0A074TY24_9MIC
O:0.025041690000000116,A0A0Q5KAR9_9MICO:0.03077753999999988):0.01349518000000
051,FGD_MICTS:0.0031248829999999117):0.018550680000000153,A0A147EU64_MICTE:0.0
0505541800000422):0.04983276999999964,A0A163G293_9MICO:0.03170618999999997):0
.1150560000000005,A0A0Q8AXV7_9MICO:0.1758001999999985):0.1039528000000007,F
GD_NAKMY:0.15074189999999987):0.06683578999999984,(((A0A0A6UL69_ACTUT:0.035434
96999999844,A0A117MS21_9ACTN:0.0422786799999999):0.04924061999999996,(R4LRU9_
9ACTN:0.03263039999999995,U5W9H0_9ACTN:0.06387586999999995):0.037922019999998
9):0.0925496300000016,R7XUN7_9ACTN:0.1922977000000013):0.04223040999999972):
0.0483142800000004,(A0A010YY07_9ACTN:0.2130304999999999,E9V3D1_9ACTN:0.205530
89999999985):0.05546503000000014):0.07330584999999967):0.1958662000000002):0.3
523835000000002):0.21869979999999956):0.1598677000000004):0.29102409999999956)
:0.7081665,E6SIZ8_THEM7:0.7081665000000003);

Data S2. MSA of F_{420}-dehydrogenases employed for ASR (fasta format)

```
>A0A147F4G7MICTE
MSVPLRFGYKASSEQFGPNELLDFGVLAEEMGFDSVFLSDHLQPWMHDGGHAPNALPWLG
ALGARTERVIIGTSVLTPTFRYHPGVIAQVFATLGVMYPGRVVLGVGTGEALNEVTLGL-
---EWPEAPERFQRLKEAITIINELWAGER-VTYEGTYYSVKDATIYDRPEQKVPIYIGA
SGPAATRLAGRIAEGYITTSGKDPELYTEKLLPALDDGLAKAGRTRDDVDTLMEVKVSYH
PDHDTALEKTRFWAPLALTAEEKMSVHDPIEMQRLANELPIERAASRFIVSTDPDEHVER
IARYVDLGFRHLVFHDPGHDQEEFLRMYGEEILPRLRKRF
>FGDMICTS
MTIPLRFGYKASSEQFGPNELLDFGVLAEEMGFDSVFLSDHLQPWMHDGGHAPNALPWLG
ALGARTERVIIGTSVLTPTFRYHPGVIAQVFATLGVMYPGRVVLGVGTGEALNEVTLGL-
---EWPEAPERFQRLKEAITIINELWAGER-VTYEGTYYSVKDATIYDRPEQKVPIYIGA
SGPAATRLAGRIAEGYITTSGKDPELYTEKLLPALDDGLSKAGRTRDDVDTLMEVKVSYH
PDHDTALEKTRFWAPLALTAEEKMSVHDPIEMQRLANELPIERAASRFIVSTDPDEHVER
IARYVDLGFRHLVFHDPGHDQEQFLRMYGEEILPRLRKRF
>A0A147EU64MICTE
MTIPLRFGYKASSEQFGPNELLDFGVLAEEVGFDSVFLSDHLQPWMHDGGHAPNALPWLG
ALGARTSRVIIGTSVLTPTFRYHPGVIAQVFATLGVMYPGRVVLGVGTGEALNEVTLGL-
---EWPEAPERFQRLKEAITIINELWAGER-VTYEGTYYSVKDATIYDRPEQKVPIYIGA
SGPAATRLAGRIAEGYITTSGKDPELYTEKLLPALDDGLGKAGRTRDDVDTLMEVKVSYH
PDHDTALEKTRFWAPLALTAEEKMSVHDPIEMQRLANELPIERAASRFIVSTDPDEHVER
IAKYVDLGFRHLVFHDPGHDQEEFLRTYGEEILPRLRKRF
>A0A074TY249MICO
MTIPLRFGYKASSEQFGPNELLEFGVLAEEMGFDSVFLSDHLQPWMHDGGHAPNALPWLG
ALGARTERVIIGTSVLTPTFRYHPGVIAQVFATLGVMYPGRVVLGVGTGEALNEVTLGL-
---DWPEPPERFQRLKEAITIINELWAGER-VTYEGTYYSVKDATIYDRPEHKVPIYIGA
SGPAATRLAGRIAEGYITTSGKDPALYTDKLLPALDDGLSKAGRTRDDVDTLMEVKVSYH
PDHDTALEKTRFWAPLALTADEKMSVHDPLEMQRLANELPIERVASRFIVSTDPDEHVER
IARYVDLGFRHLVFHDPGHDQKEFLHMYGEEILPRLRKRF
>A0A0Q6ECN29MICO
MTVPLRFGYKASSEQFGPNELLDFGVLAEKMGFDSVFLSDHLQPWMHDGGHAPNALPWLG
ALGARTERVIIGTSVLTPTFRYHPGVIAQVFATLGVMYPGRVVLGVGTGEALNEVTLGL-
---DWPEAPERFQRLKEAIAIINELWAGER-VTYEGTYYSVKDATIYDRPEQKVPIYIGA
SGPAATRLAGRIAEGYITTSGKDPALYTDTLLPALDDGLGKAGRTRDDVDTLMEVKVSYH
PDHDTALEKTRFWAPLALSADEKMSVHDPLEMQRLAAELPIERVASRFIVSTDPDEHVER
IARYVDLGFRHLVFHDPGHDQEEFLRMYGEEILPRLRKRF
>A0A0Q5KAR99MICO
MSVPLRFGYKASSEQFGPNELLDFGVMAEQVGFDSVFLSDHLQPWMHDGGHAPNALPWLG
ALGARTERVIIGTSVLTPTFRYHPGVVAQVFATLGVMYPGRVVLGVGTGEALNEVTLGL-
---EWPDPPERFQRLKEAITIINELWAGER-VTYEGTYYSVKDATIYDRPEQKVPIYIGA
SGPAATRLAGRIAEGYITTSGKDAELYTGKLLPALDDGLSKAGRTRDDVDTLMEVKVSYH
PDHDTALEKTRFWAPLALSAEEKMSVHDPLEMQRLANELPIERAASRFIVSTDPDEHVER
IARYVDLGFRHLVFHDPGHDQKQFLTMYGEEILPRLRKRF
>A0A163G2939MICO
MVVPLRFGYKASSEQFGPNELLDFSVLAEEVGFDSVFLSDHLQPWRHEGGHAPNALPWLG
ALGARTSRVILGTSVLTPTFRYHPGVIAQVFATLGVMFPGRVALGVGTGEALNEVTLGL-
---EWPEPPERFQRLKEAITLIDKLWTEDR-VTFEGTYYSVKDATIYDRPEQKVPIYIGA
SGPAATRLAGRIAEGYITTSGKDPSLYTDTLLPALDDGLAKAGRTRDDVDTLMEVKVSYH
PDRETALEKTRFWAPLALSAEEKMSVHDPIEMARLADELPIERAASRFIVSTDPDEHVER
IAHYVDLGFRHLVFHDPGHDQEEFLRTYGAEILPRLRARF
>A0A0Q8LV009MICO
MTLPLRFGYKASAEQFGPNELLDYAVLAEEVGFDSVFVSDHLQPWLHEGGHAPASVPWLG
ALGAKTSRVLIGTSVLTPTFRYNPTVVAQDFATLGVMYPGRVILGVGTGEALNEANLGI-
---AWPDPPERFQRLKEAIGLIRRLWSEDR-VNFDGTYYSARNITIYDKLPEPVPIYIGA
AGPAATRLAGRIADGFITTSGKKRELYTETLLPALHEGLEKAGRAPDAIDTLMEIKVSLD
TSLEAAQEKTRFWAPLALTPDEKMGVDDPIEMQRLGEELPIERAASRFIVSDDPDEHVER
ISWYIDLGFRHLVFHDPGHDQEAFLRRYGDEILPRLRAKY
>A0A0Q8AXV79MICO
MTIPLRFGYKASAEQFGPTELLDYAILAEEVGFDSVFISDHLQPWLHEGGHAPASVPWLG
ALGARTSKVLIGTSVLTPTFRYNPTVVAQDFATLGVLNPGRVILGVGTGEALNEANLGL-
---AWPDPPERFQRLKEAIGLIKRLWSEDR-VNFEGTYYTARNTTIYDRPEQPVPIYIGA
AGPAATRLAGRIADGFITTSGKKQELYTETLLPALREGLEKADRAPDAIDTLIEVKVSLD
RTVDAAREKTRFWAPLALTPEEKMGVDDPIEMQRLGEQLPIERTASRFIVSDDPDEHVEK
IGWYIDLGFRHLVFHDPGHDQAAFLRRYGEEILPRLRAKY
>FGDNAKMY
TPPPIRFGYKASAEQFGPSELADLAVAAEEHGFDSVFISDHLQPWRHDGGHAPASLPWLG
AVGARTQRVILGTSVLTPTIRYHPGVIAQAFATLGSMYPGRIVLGVGTGESLNEVPLGL-
```

```
---AWPEQKERFARLKESVNLIHELWTGER-VTFEGEYYRTDKATIYDRPADPVPIYIGA
SGPAATRLAGRIADGFITTSGKAPSLYTDTLLPALRDGVEKAGRRLDELDTLMEMKVSFD
TDRERAMQDTRFWAALALKPEQKAGVEDPLEMQRLADELPIEQAASRWIVSDDPDEHVEK
IRTYLDLGFRHLVFHAPGHDQQRFLALYGEQILPRLRALV
>U5W9H09ACTN
---MIRFGYKASAEQFAPRELLGYGIEAERQGFDSVFVSDHFQPWRHDGGHAPAALPWLG
ALATSTERVLIGTSVLTPTFRYHPAVVAQAFATLGCLAPGRVILGVGSGESLNEVPLGL-
---HWPDGKERFARLKEAVTLIRQLWTEDR-VTFDGTFYRTELATIYDKPEQPVPIYIGA
SGPAATRLAGRIADGFITTSGKGSELYTDTLLPAVREGAEKANRSLDDVDLMIEVKVSFD
PDLEKARNDTQYWGALALSPDEKTGVEDPVEMQRLADELPVDRTVTRWIVSSDPEEHAAK
VTEYLDMGFKHLVFHAPGPDQDRFLRVYGEEILPRLRDRV
>R4LRU99ACTN
---MIRFGYKASAEQFAPRELLRYGIDAEQQGFDSVFVSDHLQPWRHDGGHAPAALPWLG
ALATSTERVLIGTSVLTPTFRYHPAVVAQAFATLGCLAPGRVVLGVGSGESLNEVPLGL-
---TWPDGKERFARLKESVQLIKQLWTEDR-VTFEGTYYRTELATIYDKPEQPVPIYIGA
SGPAATRLAGRIADGFITTSGKGHELYTDTLLPAVKEGAEKASRSLDDLDLMIEVKVSFD
PDLERARNDTHYWGALALSPEEKTGVEDPIEMQRRADALPVDRTVTRWIVSADPDEHAAK
VAEYLDMGFKHLVFHAPGPDQDRFLRVYGEEILPRLREHK
>A0A117MS219ACTN
---MIRFGYKASAEQFAPAELLRYGILAEELGFDSVFVSDHLQPWRHDGGHAPAALPWLG
ALAARTERILVGTSVLTPTFRYHPAVVAQAFATLGCLAPGRAILGVGSGESLNEVPLGI-
---AWPDGKERFARLKEAVLLIQKLWAEDR-VTHEGQFFSTENATIYDKPETPVPIYIGA
SGPAATRMAGRIADGFITTSGKGHGLYTDTLLPAVTEGAEKAGRKIDDLDLMIEVKVSFD
DDLDRARNDTHYWGALALSPEEKTGVEDPVEMQRLADALPVERTATRWIVSSDPEEHAAK
VVEYLDMGFKHLVFHAPGPDQERFLRLYSTEILPRLRDRS
>A0A0A6UL69ACTUT
---MIRFGYKASAEQFAPAELLKYGILAEELGFDSVFVSDHLQPWRHDGGHAPAALPWLG
ALAARTEKVLIGTSVLTPTFRYHPAVVAQAFATLGCLAPGRAILGVGSGESLNEVLLGT-
---RWPDGKERFARLKEAVLLIKQLWAEDR-VTHEGQFYSTENATIYDKPEQPVPIYIGA
SGPAATRLAGRIADGFITTSGKGHELYTDTLLPAVTEGAEKAGRKIDDLDLLIEVKVSFD
DDIERARNDTHYWGALALSPEEKTGVEDPIEMQRLADALSVERTATRWIVSSDPAEHAAK
VAEYLDMGFKHLVFHAPGPDQERFLRLYSEQILPQLRSRS
>R7XUN79ACTN
---MLRFGYKASSEQFAPRELADLAVLAEDSGFDSVFISDHLQPWRHDGGHAPAALPWLG
AVAARTSRVALGTSVLTPTFRYHPGVIAQAFATLGCLAPGRIILGVGSGESLNEAPLGL-
---EWPEGKERFARLKEALQVIQSLWAGER-VSFEGDYYQLANATIYDLPEQPVPLYVAA
SGPSATRLAGRVGDGFICTSGKGRSLYVDTLLPALAEGAAKSDRTLDDLDLMIEVKVSFD
EDLSRAMDDTRFWGALALSSDEKTGVEDPIEMQRLADALPVERTASRFIVSSDPDEHVEK
VWAYVEMGFRHLVFHAPGPDQARFMAAYEKDVLPRLRARA
>A0A021VMU49CELL
---MLQFGYKASAEQFAPRELLEYGVAAERAGFDSVFVSDHLQPWRHTGGHAPAALPWLG
ALLERTERVTVGTSVLTPTFRYHPGVVAHAFATLGSLAPGRVILGIGSGEAMNEAPLGI-
---EWPDGKERFARFKEAIQLIQELWAGER-VTFEGTHYRTEQATIYDRPDERVPIYVAA
SGPAATRLAGRVGDGFITTSGKGRELYTETLLPALAEGAAKAGRSTTDMDLMMEVKVSFD
TDRARALEDTRYWGALALSSEEKTGVEDPVEMERLADALPVERTASRWIVSDDAEEHVER
VWEYVDMGFTHLVFHAPGPDQGRFIDLYREQVLPRLHARA
>A0A010YY079ACTN
---MLKFGYKASAEQFGPRELLDYAVHAEKVGFDSVWVSDHLQPWRHDGGHAPNAIAWLG
ALGASTERVQMGTSVLTPTFRYHPGLIAQVFATLGLLYPERVILGVGSGESMNEVPLGV-
---EWPDNKERFARLKEAIEIIKLLWSEDR-VDYQGQFWSLDKATIYDKPSTPVPLYVAG
SGPAATRMAGRIGDGYITTSGKAPELYTDKLLPALNEGAEKAGRDVKNLDLMIEVKVSFD
PDRAKALEDTRFWGALALKPEEKSDVEDPIEMQRLADALPTERTASRFIVSDEPEEHVAA
IKRYIDLGFTHLVFHAPGPDQARFLDLYGERILPLLRKQG
>E9V3D19ACTN
-MTEIRFGYKASNEQFAPTELLNYGVMAEEQGFDSVFISDHLQPWRHDGGHAPFAMTWLG
ALGARTERIVMGTSVLTPTFRYHPAIVAQSFATLGSLFPDRVVLGMGTGEAMNEAPLGV-
---EWPEGKVRFARFREAVRVIKKLWTEDR-VTFEGDYYQLDRATIYDKPSTPVPIYLAG
SGPAATKYAGRAGDGYITTSGKGAELYTETLLPAVRTGIELSGRKPEDVDMMIEVKVSFD
HDLEQAMEATRFWGALGLTPEQKHSVEDPVEMQRLADALPTEATAKRFIVSTDPDEHVAR
IKEYLDMGFTHLVFHAPGPDQAKFLELYGEEILPKLRALI
>A0A0F2G5349ACTN
-MTEIRFGYKASNEQFAPTELLNYGVMAEEQGFDSVFISDHLQPWRHDGGHAPFAMTWLG
ALGARTERIVMGTSVLTPTFRYHPAIVAQSFATLGSLFPDRVVLGMGTGEAMNEAPLGV-
---EWPEGKVRFARFREAVRVIKKLWTEDR-VTFEGDYYQLDRATIYDKPSTPVPIYLAG
SGPAATKYAGRAGDGYITTSGKGAELYTDTLLPAVRNGIELSGRKPEDVDMMIEVKVSFD
HDLEQAMEATRFWGALGLTPEQKHSVEDPVEMQRLADALPTEATAKRFIVSTDPDEHVAR
IKEYLDMGFTHLVFHAPGPDQAKFLELYGTEILPKLRGLV
>A0A0Q6R9Q99MICO
```

```
STVPFRIGYKASSEQFAPRELLDYGALAEQAGFESVFISDHLQPWKHTDGHAPAAIAWLG
ALGAKTERIVMGTSVLTPTFRHHPAMVAQAFGTLGQLFPGRVILGVGTGESLNEAPLGF-
---VWPDIKERFARLKEAVQLIERLWDEER-VSHEGEHYRTENVTIYDRPEVKVPIYIGA
SGPAATRLAGRIADGYITTSGKPRELYTENLLPAFREGLEKGSRAPGSVDTLIEMKVSFD
TDRQRAMEDTRHWAALALSGDEKMGVEDPREMEKLADALPLERAASRWIVSTDPDEHVER
VMDYVRMGFRHLVFHAPGPDQARFLELYGTEVVPRLRAAV
>A1T2Y6MYCVP
-MAELKLGYKASAEQFAPRELVELAVAAEEHGMDSATVSDHFQPWRHEGGHAPFSLAWMT
AVGERTKRLQLGTSVLTPTFRYNPAVIAQAFATMGCLYPGRIFLGVGTGEALNEIATGY-
-EGDWPEFKERYARLRESVRLMRDLWLGDR-VDFEGEYYKTKGASIYDVPEGGIPIYIAA
GGPQVAKYAGRAGDGFICTSGKGEELYKDKLIPAMREGAEAAGKNPDDIDRMIEIKISYD
TDPELALENTRFWAPLSLTAEQKHSIDDPIEMEKAADALPIEQVAKRWIVASDPDEAVAK
VKDYVDWGLNHLVFHAPGHDQRRFLELFRRDLEPRLRKLG
>A0A024QK85MYCNE
-MAELKLGYKASAEQFAPRELVELAVAAEEHGMDSATVSDHFQPWRHEGGHAPFSLAWMT
AVGERTQRLQLGTSVLTPTFRYNPAVIAQAFATMGCLYPGRIFLGVGTGEALNEIATGY-
-EGEWPEFKERYARLRESVRLMRELWLGDR-VDFEGEYYKTKGASIYDVPEGGIPIYIAA
GGPQVAKYAGRAGDGFICTSGKGEELYKEKLIPAMREGAEAADKNPDDVDRMIEIKISYD
TDPELALENTRFWAPLSLTAEQKHSIDDPIEMEKAADALPIEQVAKRWIVASDPDEAVAK
VKDYVDWGLNHLVFHAPGHDQRRFLELFKRDLEPRLRKLA
>A0A132SJE89MYCO
-----------------PRELVELAVAAEEHGMDSATVSDHFQPWRHQGGHAPFSLVWMT
AVGERTRRLQLGTSVLTPTFRYNPAVIAQAFATMGCLYPGRIFLGVGTGEALNEIATGY-
-EGEWPEFKERYARLRESVRLMRELWLGDR-VDFAGDYYKTKGASIYDVPEGGIPIYIAA
GGPQVAKYAGRAGDGFICTSGKGEELYKDKLIPAMREGAEAAGKNPDDVDRMIEIKISYD
TDPELALENTRFWAPLSLTAEQKHSIDDPIEMEKAADALPIEQVAKRWIVASDPDEAVAK
VKDYVDWGLNHLVFHAPGHDQRRFLELFQRDLEPRLRKLG
>V5X8A3MYCNE
-MAELKLGYKASAEQFAPRELVELAVAAEEHGMDSATVSDHFQPWRHEGGHAPFSLAWMT
AVGERTERLILGTSVLTPTFRYNPAVIAQAFATMGCLYPNRIFLGVGTGEALNEIATGY-
-EGEWPEFKERYARLRESVRLMRELWLGDR-VDFEGEYYKTKGASIYDVPEGGIPIYIAA
GGPQVAKYAGRAGDGFICTSGKGEELYKEKLIPAMREGAEAAGKNAQDIDRMIEIKISYD
TDPEAALENTRFWAPLSLTAEQKHSIDDPLEMEKAADELPIEQVAKRWIVASDPDEAVAK
VKDYVDWGLNHLVFHAPGHDQRRFLELFKRDLEPRLRKLA
>A0A0Q8ZNK99MYCO
-MAELKLGYKASAEQFAPRELVELAVEAEAHGMDSATVSDHFQPWRHEGGHAPFSLAWMT
AVGERTQRLILGTSVLTPTFRYNPAVIAQAFATMGCLYPDRIFLGVGTGEALNEIATGY-
-EGEWPEFKERYARLRESVRLMRELWLGDR-VDFEGEYYKTKGASIYDVPEGGIPIYIAA
GGPQVAKYAGRAGDGFICTSGKGEELYKEKLIPAMREGAEAADKNPDDIDRMIEIKISYD
TDPEAALENTRFWAPLSLTAEQKHSIDDPLEMEKAADELPIEQVAKRWIVASDPDEAVAK
VKDYVDWGLNHLVFHAPGHDQRRFLELFKRDLEPRLRKLG
>FGDMYCGI
-MAELKLGYKASAEQFAPRELVELAVAAEEHGMDSATVSDHFQPWRHEGGHAPFSLAWMT
AVGERTTRLQLGTSVLTPTFRYNPAVIAQAFATMGCLYPDRIFLGVGTGEALNEIATGY-
-EGEWPEFKERYARLRESVRLMRELWLGDR-VDFEGEYYKTKGASIYDVPEGGIPIYIAA
GGPQVAKYAGRAGDGFICTSGKGEELYKEKLIPAMREGAEAAGKNPDDVDRMIEIKISYD
TDPELALENTRFWAPLSLTAEQKHSIDDPMEMEKAADELPIEQVAKRWIVASDPDEAVAK
VADYVDYGLNHLVFHAPGHDQRRFLELFQRDLEPRLRKLG
>A0A0J6W3L29MYCO
-MAELKLGYKASAEQFAPRELVELAVLAEEHGMDSATVSDHFQPWRHEGGHAPFSLAWMT
AVGERTKRLVLGTSVLTPTFRYNPAVIAQAFATMGCLYPDRIFLGVGTGEALNEIATGY-
-EGEWPEFKERYARLRESVRLMRELWLGDR-VDFDGEYYKTKGASIYDVPEGGIPIYIAA
GGPQVAKYAGRAGDGFICTSGKGEELYKDKLIPAMREGAEAAGKNPDDVDRMIEIKISYD
TDPELALENTRFWAPLSLTAEQKHSIDDPMEMEKAADALPIEQVAKRWIVASDPDEAVEK
VRDYVGWGLNHLVFHAPGHDQKRFLELFQRDLEPRLRRLG
>A0A0G3IEC49MYCO
-MAELKLGYKASAEQFAPRELVELAVLAEAHGMDSATVSDHFQPWRHEGGHAPFSLAWMT
AVGERTKRLILGTSVLTPTFRYNPAVIAQAFATMGCLYPDRIFLGVGTGESLNEIATGY-
-EGEWPEFKERYARLRESVRLMRELWLGDR-VDFEGEYYKTKGASIYDVPEGGIPIYIAA
GGPQVAKYAGRAGDGFICTSGKGEELYKDKLIPAMKEGAEAAGKNPDDIDRMIEIKISYD
TDPELALENTRFWAPLSLTAEQKTNIHDPLEMEKAADELPIEQVAKRWIVASDPDEAVAK
VKDYVDWGLNHLVFHAPGHDQRRFLELFQKDLEPRLRKLG
>L0IPW19MYCO
-MAELKLGYKASAEQFAPRELVELAVAAEEHGMDSATVSDHFQPWRHEGGHAPWSLAWMA
AVGERTKRLVLGTSVLTPTFRYNPAVIAQAFATMGCLYPDRIFLGVGTGEALNEIATGY-
-EGEWPEFKERYARLRESVRLMRELWLGDR-VDFEGEFYKTKGASIYDVPEGGIPIYIAA
GGPQVAKYAGRAGDGFICTSGKGEELYKDKLIPAMREGAEAAGKDPDAVDRMIEIKISYD
TDPELALENTRFWAPLSLTAEQKHSIDDPIEMEKAADALPIEQVAKRWIVASDPDEAVAK
```

VKDYVDWGLNHLVFHAPGHDQRRFLELFAKDLEPRLRRLG
>Q9XC09MYCPH
-MAELKLGYKASAEQFAPRELVELAVAAEEHGMDSATVSDHFQPWRHEGGHAPFSLAWMT
AVGERTKRLILGTSVLTPTFRYNPAVIAQAFATMGCLYPGRVFLGVGTGEALNEIATGY-
-IGEWPEFKERFARLRESVKLMRELWLGDR-VDFEGEYYRTKGASIYDVPEGGIPVYIAA
GGPVVAKYAGRAGDGFICTSGKGEELYKDKLIPAVKEGAEAAGRNPDDVDRMIEIKISYD
TDPELALENTRFWAPLSLTAEQKHSIDDPIEMEKAADALPIEQVAKRWIVASDPDEAVEK
VKQYVDWGLNHLVFHAPGHDQRRFLELFKRDLEPRLRRLG
>A0A100X7359MYCO
-MAELKLGYKASAEQFAPRELVELAVAAEEHGMDSATVSDHFQPWRHEGGHAPFSLAWIT
AVGERTKNLVLGTSVLTPTFRYNPAVIAQAFATMSCLYPNRIFLGVGTGEALNEIATGY-
-EGDWPEFKERFARLRESVKLMRELWLGDR-VDFEGEYYRTKGASIYDVPEDGVPVYIAA
GGPVVAKYAGRAGDGFICTSGKGEELYKDKLIPAVREGAEAAGRDPDSIDRMIEIKISYD
TDPELALENTRFWAPLSLTAEQKHSIDDPIEMEKAADELPIEQVAKRWIVASDPDEAVEK
VKDYVDWGLNHLVFHAPGHDQRRFLELFQRDLEPRLRRLG
>A0A117K3969MYCO
----MKLGYKASAEQFAPRELVELAVAAEEHGMDSATVSDHFQPWRHEGGHAPFSLAWIT
AVGERTKNLLLGTSVLTPTFRYNPAVIAQAFATMSCLYPDRIFLGVGTGEALNEIATGY-
-EGDWPEFKERFARLRESVKLMRELWLGDR-VDFDGEYYRTKGASIYDVPEGGVPVYIAA
GGPVVAKYAGRAGDGFICTSGKGEELYKDKLIPAVREGAEAAGRDPDSIDRMIEIKISYD
TDPELALENTRFWAPLSLTAEQKHSIDDPIEMEKAADELPIEQVAKRWIVASDPDEAVEK
VKDYVDWGLNHLVFHAPGHDQRRFLELFQRDLEPRLRRLG
>A0A127RDL8MYCSI
-MAELKLGYKASAEQFAPRELVELAVAAEAHGMDSATVSDHFQPWRHEGGHAPFSLAWMT
AVGERTKRLQLGTSVLTPTFRYNPAVIAQAFATMGCLYPDRIFLGVGTGESLNEIATGY-
-EGEWPEFKERYARLRESVRLMRELWLGDR-VDFDGEYYHTKGASIYDVPEGGIPIYIAA
GGPQVAKYAGRAGDGFICTSGKGEELYKDKLIPAMREGAQAAGKNPDDIDRMIEIKISYD
TDPDLALENCRFWAPLSLTAEQKTSIHDPLEMEKAADELPIEQVAKRWIVASDPDEAVEK
VGQYVQWGLNHLVFHAPGHDQRRFLELFERDLAPRLRRLA
>J4SKB89MYCO
-MAELKLGYKASAEQFAPRELVELAVAAEGHGMDSATVSDHFQPWRHEGGHAPFSLAWMT
AVGERTKRLVLGTSVLTPTFRYNPAVIAQAFATMGCLYPDRIFLGVGTGESLNEIATGY-
-EGEWPEFKERYARLRESVRLMRELWLGDR-VDFEGEYYHTKGASIYDVPEGGIPIYIAA
GGPQVAKYAGRAGDGFICTSGKGEELYKDKLMPAMKEGAEAAGKNPDDIDRMIEIKISYD
TDPDKALENTRFWAPLSLTAEQKTSIHDPLEMEKAADELPIEQVAKRWIVASDPDEAVEK
VGQYVKWGLNHLVFHAPGHDQRRFLELFEKDLAPRLRRLA
>I4BDD3MYCCN
-MAELKLGYKASAEQFAPRELVELAVAAEEHGMDSATVSDHFQPWRHEGGHAPFSLAWMT
AVGERTDKLQLGTSVLTPTFRYNPAVIAQAFATMGCLYPGRIFLGVGTGESLNEIATGY-
-EGEWPDFKERYARLRESVRLMRELWLGDR-VDFDGEYYNTKGASIYDVPEGGIPIYIAA
GGPQVAKYAGRAGDGFICTSGKGEELYKDKLIPAVREGAEAAGKNPDDIDRMIEIKISYD
TDPELALENTRFWAPLSLTAEQKTNIHDPMEMEKAADELPIEQVAKRWIVASDPDEAVEK
VKDYVGWGLNHLVFHAPGHDQKRFLELFQRDLEPRLRRLA
>A0A024JU469MYCO
-MAELKLGYKASAEQFAPRELVELAVAAEAHGMDSATVSDHFQPWRHKGGHAPFSLAWMT
AVGERTERLVLGTSVLTPTFRYNPAVIAQAFATMGCLYPNRIFLGVGTGEALNEIATGY-
-EGEWPEFKERYARLRESVRLMRELWLGDR-VDFEGEYYKTKGASIYDVPEGGIPIYIAA
GGPQVAKYAGRAGDGFICTSGKGEELYKDKLIPAMREGAEAAGKNADDIDRMIEIKISYD
TDPELARENTRFWAPLSLTAEQKHSIDDPIEMEKAADALPIEQITKRWIVASDPDEAVEK
VGQYVEWGLNHLVFHAPGHDQRRFLELFEKDLAPRLRRLG
>A0A0N9X869MYCFO
-MAELKLGYKASAEQFAPRELVELAVLAEAAGMDSATVSDHFQPWRHEGGHAPFSLAWMT
AVGERTQRLVLGTSVLTPTFRYNPAVIAQAFATMGCLYPGRVFLGVGTGEALNEIATGY-
-AGEWPEFKERFARLRESVKLMRELWLGDR-VDFDGEYYKLRGASIYDVPEGGIPVYIAA
GGPVVAKYAGRAGDGFICTSGKGEELYKDKLIPAVREGAEAAGRNAEDIDRMIEIKISYD
PDPELALENTRFWAPLSLTPEQKHSIDDPIEMEKAADALPIEQVAKRWIVASDPDEAVEK
VGQYVRWGLNHLVFHAPGHDQRRFLDLFKKDLEPRLRKLG
>FGDMYCFO
-MAELKLGYKASAEQFAPRELVELAVLAEAAGMDSATVSDHFQPWRHEGGHAPFSLAWMT
AVGERTQRLVLGTSVLTPTFRYNPAVIAQAFATMGCLYPGRVFLGVGTGEALNEIATGY-
-IGQWPEFKERFARLRESVKLMRELWLGDR-VDFDGEYYKLRGASIYDVPEGGIPVYIAA
GGPVVAKYAGRAGDGFICTSGKGEELYKDKLIPAVREGAEAAGRNAEDIDRMIEIKISYD
PDPELALENTRFWAPLSLTPEQKHSIDDPIEMEKAADALPIEQVAKRWIVASDPDEAVEK
VGQYVTWGLNHLVFHAPGHDQRRFLDLFKKDLEPRLRKLG
>A0A0K0WZZ69MYCO
-MAELKLGYKASAEQFAPRELVELAVLAESAGMDSATVSDHFQPWRHEGGHAPFSLAWMT
AVGERTQRLVLGTSVLTPTFRYNPAVIAQAFATMGCLYPGRIFLGVGTGEALNEIATGY-
-AGEWPEFKERFARLRESVKLMRELWLGDR-VDFDGEYYHTKGASIYDVPEGGIPVYVAA

GGPVVAKYAGRAGDGFICTSGKGEELYAEKLIPAVKEGAEAAGRHADDIDRMIEIKISYD
TDPEAALENTRFWAPLSLTAEQKHSIDDPIEMEKAADALPIEQVAKRWIVASDPDEAVEK
VGQYVKWGLNHLVFHAPGHDQRRFLELFKRDLEPRLRKLA
>X5L6I09MYCO
LVAELKLGYKASAEQFAPRELVELAVLAEASGMDSATVSDHFQPWRHEGGHAPFSLAWMT
AVGERTERLVLGTSVLTPTFRYNPAVIAQAFATMACLYPGRIFLGVGTGEALNEIATGY-
-AGEWPEFKERFARLRESVKLMRELWLGDR-VDFDGEYYRLKGASIYDVPEGGVPVYIAA
GGPVVAKYAGRAGDGFICTSGKGEELYKDKLIPAVKEGAEAAGRNADDIDRMIEIKISYD
PDPKLALENTRFWAPLSLTAEQKHSIDDPIEMEKAADGLPIEQVAKRWIVASDPDEAVAK
VKDYVDWGLNHLVFHAPGHDQRRFLDLFKTDLEPRLRKLG
>K5BH82MYCHD
--MELKLGYKASAEQFGPRELVELAVAAEAHGMDSATVSDHFQPWRHKGGHAPFSLAWMA
AVGERTKKLILGTSVLTPTFRYNPAVIAQAFATMGCLYPGRVFLGVGTGEALNEIATGF-
-EGEWPEFKERFARLRESVRLMRALWAGDR-VDFEGEYYRTKGASIYDVPPDGIPVYIAA
GGPVVAKYAGRAGDGFICTSGKGEELYKDKLIPAVKEGAEAAGRNPDDIDRMIEIKISYD
PDPELALNNTRFWAPLSLTAEQKHSIDDPIEMEKAADALPIEQVAKRWIVASDPDEAVEQ
VRQYVDWGLNHLVFHAPGHDQRRFLELFKRDLEPRLRRLG
>A0A0B2YM229MYCO
-MAELKLGYKASAEQFAPRELVELAVAAEAAGMDSATVSDHFQPWRHEGGHAPFSLAWMT
AVGERTERLVLGTSVLTPTFRYNPAVIAQAFATMGCLYPDRIFLGVGTGEALNEIATGY-
-EGDWPEFKERYARLRESVKLMRELWLGDR-VDFEGEYYKTKGASIYDVPEGGIPIYIAA
GGPQVAKYAGRAGDGFICTSGKGEELYKDKLIPAVKEGAEAAGRNAEDIDRMIEIKISYD
PDPQKALENTRFWAPLSLTAEQKHSINDPIEMEKAADELPIEQVAKRWIVASDPDEAVEM
VGQYVKWGLNHLVFHDPGHDQRRFLELFKNDLEPRLRKLG
>A0A0D1L1H49MYCO
-MAELRLGYKASAEQFAPRELVELAVAAEAAGMDSATVSDHFQPWRHTGGHAPFSLAWMT
AVGERTERLLLGTSVLTPTFRYNPAVIAQAFATMGCLYPDRIFLGIGTGEALNEIATGY-
-EGEWPEFKERYARLRESVRLMRELWNGDR-VDFDGEYYKTKGASIYDVPEGGIPIYIAA
GGPQVAKYAGRAGDGFICTSGKGEELYKDKLIPAMREGAEAAGKNPDDVDRMIEIKISYD
PDPALALENTRFWAPLSLTAEQKHSIEDPIEMEAAADALPIEQVAKRWIVASDPDEAVEK
VKQYVDWGLNHLVFHDPRQDQRRFLELFKTDLEPRLRKLG
>V7NPB8MYCAV
-MAELKLGYKASAEQFAPRELVELAVAAEAHGMDSATVSDHFQPWRHEGGHAPFSLAWMT
AVGERTTRITLGTSVLTPTFRYNPAVVAQAFATMACLYPGRIFLGVGTGEALNEIATGY-
-QGEWPEFKERFARLRESVRLMRELWRGDR-VDFDGEYYRLKGASIYDVPDGGVPIYIAA
GGPAVAKYAGRAGDGFICTSGKGEELYKDKLIPAVKEGAAINDRNVDDIDKMIEIKISYD
PDPELALENTRFWAPLSLTAEQKHSIDDPIEMEKAADALPIEQVAKRWIVASDPDEAVAK
VKDYVDWGLNHLVFHAPGHDQRRFLELFEKDLAPRLRRLG
>A0A0E2WNU7MYCAV
-MAELKLGYKASAEQFAPRELVELAVAAEAHGMDSATVSDHFQPWRHEGGHAPFSLAWMT
AVGERTTRITLGTSVLTPTFRYNPAVVAQAFATMACLYPGRIFLGVGTGEALNEIATGY-
-QGEWPEFKERFARLRESVRLMRELWRGDR-VDFDGEYYRLKGASIYDVPDGGVPIYIAA
GGPAVAKYAGRAGDGFICTSGKGEELYKDKLIPAVKEGAAINDRNVDDIDKMIEIKISYD
PDPELALENTRFWAPLSLTAEQKHSIDDPIEMEKAADALPIEQVAKRWIVASDPDEAVAK
VKDYVDWGLNHLVFHAPGHDQRRFLELFEKDLAPRLRRLG
>Q6R2X5MYCPC
-MAELKLGYKASAEQFAPRELVELAVAAEAHGMDSATVSDHFQPWRHEGGHAPFSLAWMT
AVGERTTRITLGTSVLTPTFRYNPAVVAQAFATMACLYPGRIFLGVGTGEALNEIATGY-
-QGEWPEFKERFARLRESVRLMRELWRGDR-VDFDGEYYRLKGASIYDVPDGGVPIYIAA
GGPAVAKYAGRAGDGFICTSGKGEELYKDKLIPAVKEGAAINDRNVDDIDKMIEIKISYD
PDPELALENTRFWAPLSLTAEQKHSIDDPIEMEKAADALPIEQVAKRWIVASDPDEAVAK
VKDYVDWGLNHLVFHAPGHDQRRFLELFEKDLAPRLRRLG
>A0A0E2WGP5MYCAV
-MAELKLGYKASAEQFAPRELVELAVAAEAHGMDSATVSDHFQPWRHEGGHAPFSLAWMT
AVGERTTRITLGTSVLTPTFRYNPAVVAQAFATMACLYPGRIFLGVGTGEALNEIATGY-
-QGEWPEFKERFARLRESVRLMRELWRGDR-VDFDGEYYRLKGASIYDVPDGGVPIYIAA
GGPAVAKYAGRAGDGFICTSGKGEELYKDKLIPAVKEGAAINDRNVDDIDKMIEIKISYD
PDPELALENTRFWAPLSLTAEQKHSIDDPIEMEKAADALPIEQVAKRWIVASDPDEAVAK
VKDYVDWGLNHLVFHAPGHDQRRFLELFEKDLAPRLRRLG
>A0A049DNN8MYCAV
-MAELKLGYKASAEQFAPRELVELAVAAEAHGMDSATVSDHFQPWRHEGGHAPFSLAWMT
AVGERTTRITLGTSVLTPTFRYNPAVVAQAFATMACLYPGRIFLGVGTGEALNEIATGY-
-QGEWPEFKERFARLRESVRLMRELWRGDR-VDFDGEYYRLKGASIYDVPDGGVPIYIAA
GGPAVAKYAGRAGDGFICTSGKGEELYKDKLIPAVKEGAAINDRNVDDIDKMIEIKISYD
PDPELALENTRFWAPLSLTAEQKHSIDDPIEMEKAADALPIEQVAKRWIVASDPDEAVAK
VKDYVDWGLNHLVFHAPGHDQRRFLELFEKDLAPRLRRLG
>FGDMYCA1
-MAELKLGYKASAEQFAPRELVELAVAAEAHGMDSATVSDHFQPWRHEGGHAPFSLAWMT

```
AVGERTTRITLGTSVLTPTFRYNPAVVAQAFATMACLYPGRIFLGVGTGEALNEIATGY-
-QGEWPEFKERFARLRESVRLMRELWRGDR-VDFDGEYYRLKGASIYDVPDGGVPIYIAA
GGPAVAKYAGRAGDGFICTSGKGEELYKDKLIPAVKEGAAINDRNVDDIDKMIEIKISYD
PDPELALENTRFWAPLSLTAEQKHSIDDPIEMEKAADALPIEQVAKRWIVASDPDEAVAK
VKDYVDWGLNHLVFHAPGHDQRRFLELFEKDLAPRLRRLG
>V7MV86MYCAV
-MAELKLGYKASAEQFAPRELVELAVAAEAHGMDSATVSDHFQPWRHEGGHAPFSLAWMT
AVGERTTRITLGTSVLTPTFRYNPAVVAQAFATMACLYPGRIFLGVGTGEALNEIATGY-
-QGEWPEFKERFARLRESVRLMRELWRGDR-VDFDGEYYRLKGASIYDVPDGGVPIYIAA
GGPAVAKYAGRAGDGFICTSGKGEELYKDKLIPAVKEGAAINDRNVDDIDKMIEIKISYD
PDPELALENTRFWAPLSLTAEQKHSIDDPIEMEKAADALPIEQVAKRWIVASDPDEAVAK
VKDYVDWGLNHLVFHAPGHDQRRFLELFEKDLAPRLRRLG
>V7IYU4MYCAV
-MAELKLGYKASAEQFAPRELVELAVAAEAHGMDSATVSDHFQPWRHEGGHAPFSLAWMT
AVGERTTRITLGTSVLTPTFRYNPAVVAQAFATMACLYPGRIFLGVGTGEALNEIATGY-
-QGEWPEFKERFARLRESVRLMRELWRGDR-VDFDGEYYRLKGASIYDVPDGGVPIYIAA
GGPAVAKYAGRAGDGFICTSGKGEELYKDKLIPAVKEGAAINDRNVDDIDKMIEIKISYD
PDPELALENTRFWAPLSLTAEQKHSIDDPIEMEKAADALPIEQVAKRWIVASDPDEAVAK
VKDYVDWGLNHLVFHAPGHDQRRFLELFEKDLAPRLRRLG
>V7KG82MYCPC
-MAELKLGYKASAEQFAPRELVELAVAAEAHGMDSATVSDHFQPWRHEGGHAPFSLAWMT
AVGERTTRITLGTSVLTPTFRYNPAVVAQAFATMACLYPGRIFLGVGTGEALNEIATGY-
-QGEWPEFKERFARLRESVRLMRELWRGDR-VDFDGEYYRLKGASIYDVPDGGVPIYIAA
GGPAVAKYAGRAGDGFICTSGKGEELYKDKLIPAVKEGAAINDRNVDDIDKMIEIKISYD
PDPELALENTRFWAPLSLTAEQKHSIDDPIEMEKAADALPIEQVAKRWIVASDPDEAVAK
VKDYVDWGLNHLVFHAPGHDQRRFLELFEKDLAPRLRRLG
>X8B6F8MYCAV
-MAELKLGYKASAEQFAPRELVELAVAAEAHGMDSATVSDHFQPWRHEGGHAPFSLAWMT
AVGERTTRITLGTSVLTPTFRYNPAVVAQAFATMACLYPGRIFLGVGTGEALNEIATGY-
-QGEWPEFKERFARLRESVRLMRELWRGDR-VDFDGEYYRLKGASIYDVPDGGVPIYIAA
GGPAVAKYAGRAGDGFICTSGKGEELYKDKLIPAVKEGAAINDRNVDDIDKMIEIKISYD
PDPELALENTRFWAPLSLTAEQKHSIDDPIEMEKAADALPIEQVAKRWIVASDPDEAVAK
VKDYVDWGLNHLVFHAPGHDQRRFLELFEKDLAPRLRRLG
>X7UM259MYCO
-MAELKLGYKASAEQFAPRELVELAVAAEAHGMDSATVSDHFQPWRHEGGHAPFSLAWMT
AVGERTTRITLGTSVLTPTFRYNPAVVAQAFATMACLYPNRIFLGVGTGEALNEIATGY-
-QGEWPEFKERFARLRESVRLMRELWRGDR-VDFDGEYYRLKGASIYDVPDGGVPIYIAA
GGPAVAKYAGRAGDGFICTSGKGEELYKDKLIPAVKEGAAINDRNVDDIDKMIEIKISYD
PDPELALENTRFWAPLSLTAEQKHSIDDPIEMEKAADALPIEQVAKRWIVASDPDEAVAK
VKDYVDWGLNHLVFHAPGHDQRRFLELFEKDLAPRLRRLG
>T2GXG4MYCHT
-MAELKLGYKASAEQFAPRELVELAVAAEAHGMDSATVSDHFQPWRHEGGHAPFSLAWMT
AVGERTQRITLGTSVLTPTFRYNPAVVAQAFATMACLYPNRIFLGVGTGEALNEIATGY-
-QGEWPEFKERFARLRESVRLMRELWRGDR-VDFDGEYYRLKGASIYDVPDGGVPIYIAA
GGPAVAKYAGRAGDGFICTSGKGEELYKDKLIPAVKEGAAINDRNVDDIDKMIEIKISYD
PDPELALENTRFWAPLSLTAEQKHSIDDPIEMEKAADALPIEQVAKRWIVASDPDEAVAK
VKDYVDWGLNHLVFHAPGHDQRRFLELFEKDLAPRLRRLG
>V7K5R9MYCAV
-MAELKLGYKASAEQFAPRELVELAVAAEAHGMDSATVSDHFQPWRHEGGHAPFSLAWMT
AVGERTTRITLGTSVLTPTFRYNPAVVAQAFATMACLYPGRIFLGVGTGEALNEIATGY-
-QGEWPEFKERFARLRESVRLMRELWRGDR-VDFDGEYYRLKGASIYDVPDGGVPIYIAA
GGPAVAKYAGRAGDGFICTSGKGEELYKDKLIPAVMEGAAINDRNVDDIDKMIEIKISYD
PDPELALENTRFWAPLSLTAEQKHSIDDPIEMEKAADALPIEQVAKRWIVVSDPDEAVAK
VKDYVDWGLNHLVFHAPGHDQRRFLELFEKDLAPRLRRLG
>V7L3J0MYCAV
-MAELKLGYKASAEQFAPRELVELAVAAEAHGMDSATVSDHFQPWRHEGGHAPFSLAWMT
AVGERTTRITLGTSVLTPTFRYNPAVVAQAFATMACLYPGRIFLGVGTGEALNEIATGY-
-QGEWPEFKERFARLRESVRLMRELWRGDR-VDFDGEYYRLKGASIYDVPDGGVPIYIAA
GGPAVAKYAGRAGDGFICTSGKGEELYKDKLIPAVMEGAAINDRNVDDIDKMIEIKISYD
PDPELALENTRFWAPLSLTAEQKHSIDDPIEMEKAADALPIEQVAKRWIVASDPDEAVAK
VKDYVDWGLNHLVFHAPGHDQRRFLELFEKDLAPRLRRLG
>A0A0U0WAL19MYCO
-MAELKLGYKASAEQFAPRELVELAVAAEGHGMDSATVSDHFQPWRHEGGHAPFSLAWMT
AVGERTKRVQLGTSVLTPTFRYNPAVIAQAFATMACLYPNRVFLGVGTGEALNEIATGY-
-QGEWPEFKERFARLRESVRLMRELWRGDR-VDFDGEYYRLKGASIYDVPEGGVPIYVAA
GGPAVAKYAGRAGDGFICTSGKGEELYAEKLIPAVKEGAAAADRNVDDIDKMIEIKISYD
PDPELALENTRFWAPLSLTAEQKHSIDDPIEMEKAADALPIEQVAKRWIVASDPDEAVEK
VGQYVGWGLNHLVFHAPGHDQRRFLDLFEKDLAPRLRRLG
```

>I2AK149MYCO
-MAELKLGYKASAEQFAPRELVELAVAAEGHGMDSATVSDHFQPWRHEGGHAPFSLAWMT
AVGERTKRITLGTSVLTPTFRYNPAVIAQAFATMACLYPGRIFLGVGTGEALNEIATGY-
-EGDWPEFKERFARLRESVRLMRELWRGDR-VDFDGEYYRLKGASIYDVPEGGVPIYVAA
GGPAVAKYAGRAGDGFICTSGKGEELYKDKLIPAVKEGAAINDRNIDDIDKMIEIKISYD
PDPKLALENTRFWAPLSLTAEQKHSIDDPIEMEKAADALPIEQVAKRWIVASDPDEAVEK
VGQYVKWGLNHLVFHAPGHDQRRFLDLFEKDLAPRLRRLA
>S4ZLU29MYCO
-MAELKLGYKASAEQFAPRELVELAVAAEGHGMDSATVSDHFQPWRHEGGHAPFSLAWMT
AVGERTKRITLGTSVLTPTFRYNPAVIAQAFATMACLYPDRIFLGVGTGEALNEIATGY-
-EGDWPEFKERFARLRESVRLMRELWRGDR-VDFDGEYYRLKGASIYDVPEGGVPIYVAA
GGPAVAKYAGRAGDGFICTSGKGEELYKDKLIPAVKEGAAINDRNVDDIDKMIEIKISYD
PDPKLALENTRFWAPLSLTAEQKHSIDDPIEMEKAADALPIEQVAKRWIVASDPDEAVEK
VGQYVKWGLNHLVFHAPGHDQRRFLDLFEKDLAPRLRRLA
>A0A081I3N89MYCO
-MAELKLGYKASAEQFAPRELVELAVAAEGHGMDSATVSDHFQPWRHEGGHAPFSLAWMT
AVGERTKRITLGTSVLTPTFRYNPAVIAQAFATMACLYPDRIFLGVGTGEALNEIATGY-
-EGDWPEFKERFARLRESVRLMRELWRGDR-VDFDGEYYRLKGASIYDVPEGGVPIYVAA
GGPAVAKYAGRAGDGFICTSGKGEELYKDKLIPAVKEGAAINDRNVDDIDKMIEIKISYD
PDPKLALENTRFWAPLSLTAEQKHSIDDPIEMEKAADALPIEQVAKRWIVASDPDEAVEK
VGQYVKWGLNHLVFHAPGHDQRRFLDLFEKDLAPRLRRLA
>A0A0U1D7K19MYCO
-MAELKLGYKASAEQFAPRELVELAVAAEGHGMDSATVSDHFQPWRHEGGHAPFSLAWMT
AVGERTERIVLGTSVLTPTFRYNPAVVAQAFATMACLYPDRIFLGVGTGEALNEIATGY-
-EGEWPEFKERFARLRESVRLMRELWRGDR-VDFDGEYYRLKGASIYDVPQGGVPIYIAA
GGPAVAKYAGRAGDGFICTSGKGEELYQDKLMPAVQEGAAAAERNVDDIDKMIEIKISYD
PDPELALENTRFWAPLSLTAEQKHSIDDPIEMEKAADALPIEQVAKRWIVASDPDEAVEK
VKQYVDWGLNHLVFHAPGHDQRRFLELFEKDLAPRLRRLA
>A0A0Q2LLI1MYCGO
-MAELKLGYKASAEQFAPRELVELAVLAEGHGMDSATVSDHFQPWRHKGGHAPFSLAWMT
AVGERTERLQLGTSVLTPTFRYNPAVIAQAFATMACLYPGRVFLGVGTGEALNEIATGY-
-EGDWPDFKERFARLRESVRLMRELWRGDR-VDFDGEYYRTKGASIYDVPEGGVPVYIAA
GGPAVAKYAGRAGDGFICTSGKGEELYTEKLIPAVKEGAAINERNIDDIDRMIEIKISYD
TDPDLALENTRFWAPLSLSAEQKHSIDDPIEMEKAADALPIEQIAKRWIVASDPDEAVEK
VRQYVTWGLNHLVFHAPGHDQRRFLELFEKDLAPRLRRLG
>A0A0J8UVG79MYCO
-MAELKLGYKASAEQFGPRELVELGVAAEAHGMDSATVSDHFQPWRHKGGHAPFSLSWMT
AVGERTERIQLGTSVLTPTFRYNPAVVAQAFATMACLYPGRVFLGVGTGEALNEIATGY-
-EGAWPQFKERFARLREAVRLMRELWRGDR-VDFDGDYYHLKGASIYDVPEGGVPIYIAA
GGPAVAKYAGRAGDGFICTSGKGEELYRDKLIPAVREGAGAADRNLDDIDKMIEIKISYD
PDPELALENTRFWAPLSLTAEQKHSIDDPIEMEKAADALPIDQVAKRWIVASDPDEAVEK
VRAYLDYGLNHLVFHAPGHDQRRFLRLFEKDLAPRLRRLA
>I0REJ9MYCXE
-MAALKLGYKASAEQFGPRELVELGVAAEAHGMDSATVSDHFQPWRHKGGHAPFSLSWMT
AVGERTERIQLGTSVLTPTFRYNPAVVAQAFATMACLYPGRVFLGVGTGEALNEIATGY-
-EGAWPEFKERFARLREAVRLMRELWRGDR-VDFDGDYYHLKGASIYDVPEGGVPIYVAA
GGPAVAKYAGRAGDGFICTSGKGEELYRDKLIPAVQEGAAAADRNLDDIDKMIEIKISYD
PDPELALENTRFWAPLSLTAEQKHSIDDPIEMEKAADALPIEQVAKRWIVASDPDEAVEQ
VRAYLDYGLNHLVFHAPGHDQRRFLRLFETDLAPRLRRLA
>A0A164E6D4MYCKA
-MAELKLGYKASAEQFAPRELVELAVAAEAHGMDSATVSDHFQPWRHQGGHAPFSLSWMT
AVGERTTRLLLGTSVLTPTFRYNPAVIAQAFATMGCLYPGRVFLGVGTGEALNEIATGY-
-EGVWPEFKERFARLRESVRLMRELWSRGR-VDFDGEYYRLKGASIYDVPEGGVPVYIAA
GGPAVAKYAGRAGDGFICTSGKGEELYTEKLIPGVKEGAAAAGRDVDDIDKMIEIKISYD
PDPELALQNTRFWAPLSLTAEQKHSIDDPIEMEKAADALPIEQIAKRWIVASDPDEAVEK
VGQYVTWGLNHLVFHAPGHDQRRFLQLFETDLAPRLRRLG
>FGDNOCFA
-MGDLELGFKASAEQFGPRELVDIAVLAEEHGMDSATVSDHFQPWRHKGGHAPFSLAWMA
AVGARTERIKLGTSVLTPTFRYNPAVIAQAFATMGCLYPGRVMLGVGTGEALNEIATGY-
-QGEWPEFKERFARLREAVELMRALWTGDR-VDFDGQYYRTVGASIYDVPEGGVPIYIAA
GGPLVARYAGRAGDGFICTSGKGMELYTDKLMPAVAEGAAKAGRSVDSIDRMIEIKISYD
TDPELALENTRFWAPLSLTAEQKHSITDPIEMEAAADALPIEQIAKRWIVASDPDQAVEQ
IKPYLDAGLNHLVFHAPGHDQRRFLDLFQRDLAPRLRALA
>A0A0H5PGI5NOCFR
-MGDLELGFKASAEQFGPRELVDIAVLAEEHGMDSATVSDHFQPWRHKGGHAPFSLAWMA
AVGARTERIKLGTSVLTPTFRYNPAVIAQAFATMGCLYPGRVMLGVGTGEALNEIATGY-
-QGEWPKFKERFARLREAVELMRALWTGDR-VDFDGQYYRTVGASIYDVPEGGVPIYIAA
GGPLVARYAGRAGDGFICTSGKGMELYTDKLMPAVAEGAAKAGRSVDSIDRMIEIKISYD

TDPELALENTRFWAPLSLTAEQKHSITDPIEMEAAADALPIEQIAKRWIVASDPDQAVEQ
IKPYLDAGLNHLVFHAPGHDQRRFLDLFQRDLAPRLRALA
>A0A0C1AY679NOCA
-MGELKLGYKASAEQFGPRELVEIAVLAEEHGLDSASVSDHFQPWRHKGGHAPFSLAWMA
AVGERTKRIQLGTSVLTPTFRYNPAVIAQAFATMGCLYPERVMLGVGTGEALNEIATGY-
-KGDWPDFKERFARLREAVELMRALWTGDR-VDFQGEYYNTVGASIYDVPKGGIPVYVAA
GGPLVARYAGRAGDGFICTSGKGMDLYTDKLMPAVAEGAAKAGRTVDDIDRMIEIKISYD
TDPELALENTRFWAPLSLTPEQKHSITDPIEMEAAADALPIEQIAKRWIVASDPDQAVDL
IKPYLDAGLNHLVFHAPGHDQRRFLDLFQRDLAPRLRALA
>A0A034UHX49NOCA
-MGELKLGYKASAEQFGPRELVEIAVLAEEHGLDSASVSDHFQPWRHKGGHAPFSLAWMA
AVGERTKRIQLGTSVLTPTFRYNPAVIAQAFATMGCLYPERVMLGVGTGEALNEIATGY-
-KGDWPDFKERFARLREAVELMRALWTGDR-VDFQGEYYNTVGASIYDVPKGGIPVYVAA
GGPLVARYAGRAGDGFICTSGKGMDLYTDKLMPAVAEGAAKAGRTVDDIDRMIEIKISYD
TDPELALENTRFWAPLSLTPEQKHSITDPIEMEAAADALPIEQIAKRWIVASDPDQAVDL
IKPYLDAGLNHLVFHAPGHDQRRFLDLFQRDLAPRLRALA
>K0FE219NOCA
-MGELKLGYKASAEQFGPRELVEIAVLAEEHGLDSASVSDHFQPWRHKGGHAPFSLAWMA
AVGERTKRIQLGTSVLTPTFRYNPAVIAQAFATMGCLYPERVMLGVGTGEALNEIATGY-
-KGDWPDFKERFARLREAVELMRALWTGDR-VDFQGEYYNTVGASIYDVPKGGIPVYVAA
GGPLVARYAGRAGDGFICTSGKGMDLYTDKLMPAVAEGAAKAGRTVDDIDRMIEIKISYD
TDPELALENTRFWAPLSLTPEQKHSITDPIEMEAAADALPIEQIAKRWIVASDPDQAVDL
IKPYLDAGLNHLVFHAPGHDQRRFLDLFQRDLAPRLRALA
>A0A0B8N8Q49NOCA
-MKDLKLGYKASAEQFGPRELVELAVAAEAHGLDSATVSDHFQPWRHNGGHAPFSLAFLA
AVGERTKRIQLGTSVLTPTFRYNPAVIAQAFATMGCLYPDRIMLGVGSGEALNEIATGY-
-TGEWPEFKERFARLRESVDLMRALWTGER-VDFDGQYYKTVGASIYDVPKGGIPVYIAA
GGPLVARYAGRAGDGFICTSGKGMDLYTEKLMPAVAEGAAKVGKTVEDIDRMIEIKISYD
TDPELALENTRFWAPLSLTAEQKHSITDPIEMEAAADALPIEQIAKRWIVASDPDQAVEQ
IKPYLDAGLNHLVFHAPGHDQRRFLDLFQRDLAPRLRAL-
>M2WXX69NOCA
--MALKLGYKASAEQFGPRELVELAVLAEERGMDSATVSDHFQPWRHKGGHAPFSLAWMT
AVGERTERIQLGTSVLTPTFRYNPAVIAQAFATMGCLYPGRVMLGVGTGEALNEIATGY-
-SGQWPEFKERFARLRESVRLMRELWSGDR-VDFEGEFYSTKGASIYDVPEGGIPVYVAA
GGPVVARYAGRAGDGFICTSGKGMELYTEKLMPAVAEGAEKAGRDVGGIDRMIEIKISYD
TDPELALENTRFWAPLSLTPEQKHSIEDPIEMEAAADALPIEQVAKRWIVASDPDEAVGR
VKEYVDAGLNHLVFHAPGHDQRRFLELFERDLAPRLRALV
>A0A0K2YIJ99NOCA
--MALKLGYKASAEQFGPRELVELAVLAESRGMDSATVSDHFQPWRHNGGHAPFSLAWMT
AVGERTSTLQLGTSVLTPTFRYNPAVIAQAFATMGCLYPGRVMLGVGTGEALNEIATGY-
-SGEWPEFKERFARLRESVRLMRELWTGDR-VDFEGEFYKTVGASIYDVPEGGIPVYIAA
GGPVVARYAGRAGDGFICTSGKGMDLYTEKLMPAVAEGAEKAGRDVAGIDXMIEIKISYD
TDPELALENTRFWAPLSLSPEQKHSIEDPIEMEAAADALPIEQVAKRWIVASDPDDAVEQ
VKAYVDAGLNHLVFHAPGHDQRRFLELFERDLAPRLRALA
>W4A0K4RHORH
MSQGLKLGYKASAEQFGPRELVELAVLAEEHGMDSATVSDHFQPWRHNGGHAPFSLAWMT
AVGERTSRLQLGTSVLTPTFRYNPAVIAQAFATMGCLYPGRVMLGVGTGEALNEIATGY-
-AGVWPEFKERFARLRESVALMRELWTGER-VDFDGEYYTTKGASIYDVPEGGIPVYIAA
GGPVVARYAGRAGDGFICTSGKGMELYTEKLLPAVAEGAGKAGRDAGAIDKMIEIKISYD
TDPERALENTRFWAPLSLTAEQKHSIDDPVQMEAAADALPIEQVARRWIVASDPDEAVEK
VAAYVDAGLNHLVFHAPGHDQRRFLDLFEKDLAPRLRALA
>A0A059MIF19NOCA
MSQGLKLGYKASAEQFGPRELVELAVLAEEHGMDSATVSDHFQPWRHNGGHAPFSLAWMT
AVGERTSRLQLGTSVLTPTFRYNPAVIAQAFATMGCLYPGRVMLGVGTGEALNEIATGY-
-AGVWPEFKERFARLRESVALMRELWTGER-VDFEGEYYTTKGASIYDVPEGGIPVYIAA
GGPVVARYAGRAGDGFICTSGKGMELYTGKLLPAVAEGAGKAGRDAGAIDKMIEIKISYD
TDPERALENTRFWAPLSLTAEQKHSIDDPMQMEAAADALPIEQVAKRWIVASDPDEAVEK
VAAYVDAGLNHLVFHAPGHDQRRFLDLFEKDLAPRLRALA
>W3ZYN4RHORH
MSQGLKLGYKASAEQFGPRELVELAVLAEKHGMDSATVSDHFQPWRHNGGHAPFSLAWMT
AVGERTSRLQLGTSVLTPTFRYNPAVIAQAFATMGCLYPGRVMLGVGTGEALNEIATGY-
-AGVWPEFKERFARLRESVALMRELWTGER-VDFEGEYYTTKGASIYDVPAGGIPVYIAA
GGPVVARYAGRAGDGFICTSGKGMELYTGKLLPAVAEGAGKAGRDAGAIDKMIEIKISYD
TDPERALENTRFWAPLSLTAEQKHSIDDPVQMEAAADALPIEQVAKRWIVASDPDEAVEK
VAAYVDAGLNHLVFHAPGHDQRRFLDLFERDLAPRLRALA
>A0A059MM579NOCA
MSQGLKLGYKASAEQFGPRELVELAVLAEEHGMDSATVSDHFQPWRHNGGHAPFSLAWMT
AVGERTSRLQLGTSVLTPTFRYNPAVIAQAFATMGCLYPGRVMLGVGTGEALNEIATGY-

```
-AGVWPEFKERFARLRESVALMRELWTGER-VDFDGEYYTTKGASIYDVPAGGIPVYIAA
GGPVVARYAGRAGDGFICTSGKGMELYTGKLLPAVAEGAGKAGREVGGIDKMIEIKISYD
TDPERALENTRFWAPLSLTAEQKHSIDDPVQMEAAADALPIEQVAKRWIVASDPDEAVEK
VAAYVDAGLNHLVFHAPGHDQRRFLDLFERDLAPRLRALA
>A0A076EZM4RHOOP
---MIKFGYKASAEQFGPRELVELGVLAEAHGMDSATVSDHFQPWRHEGGHAPFSLAWMT
AVGERTSRLQLGTSVMTPTFRYNPAVVAQAFATMGCLYPGRIMLGVGTGEALNEIATGF-
-AGEWPEFKERFARLREAVRLMRELWLGDR-VDFEGEYFTTKGASIYDVPEGGIPVYIAA
GGPVVARYAGRSGDGFICTSGKGMELYTEKLMPAVAEGAAKAGRDVAEIDKMIEIKISYD
TDPELALENTRFWAPLSLTPEQKHSIDDPIEMEKAADALPIEQVAKRWIVASDPDEAVAQ
IRPYLDAGLNHLVFHAPGHDQKRFLELFERDLAPRLRGLA
>A0A034TYZ89NOCA
-MTELRLGYKASAEQFAPRELVELTVSAEENGFDSAMISDHFQPWRFNGGHAPFSLAWLA
AVGERTRRIQIGTSVLTPTFRYNPAVIAQAFATMACLYPGRVMLGVGSGEALNEIATGF-
-IGEWPEFKERYARLRESVELMRALWTGDR-VDFDGEYYKTVGASIYDVPAEGVPVYIAA
GGPLVARYAGRVGDGFICTSGKGMDLYTEKLMPAVTEGAEKAGRTVADVDTMIEIKLSYE
TDYDLALQNTRFWAPLSLTPEQKHSISDPIEMEAAADALPIEQIAKRWIVGTDPDEVVSR
IEPYIDAGMRHLVFHAPGHDQSRFLELFARDLAPRLRKLG
>A0A0C1CMG39NOCA
-MTELRLGYKASAEQFAPRELVELTVSAEENGFDSAMISDHFQPWRFNGGHAPFSLAWLA
AVGERTRRIQIGTSVLTPTFRYNPAVIAQAFATMACLYPGRVMLGVGSGEALNEIATGF-
-IGEWPEFKERYARLRESVELMRALWTGDR-VDFDGEYYKTVGASIYDVPAEGVPVYIAA
GGPLVARYAGRVGDGFICTSGKGMDLYTEKLMPAVTEGAEKAGRTVADVDTMIEIKLSYE
TDYDLALQNTRFWAPLSLTPEQKHSISDPIEMEAAADALPIEQIAKRWIVGTDPDDVVSR
IEPYIDAGMRHLVFHAPGHDQSRFLELFARDLAPRLRKLG
>K0F4H79NOCA
-MTELRLGYKASAEQFAPRELVELTVSAEENGFDSAMISDHFQPWRFNGGHAPFSLAWLA
AVGERTRRIQIGTSVLTPTFRYNPAVIAQAFATMACLYPGRVMLGVGSGEALNEIATGF-
-IGEWPEFKERYARLRESVELMRALWTGDR-VDFDGEYYKTVGASIYDVPAEGVPVYIAA
GGPLVARYAGRVGDGFICTSGKGMDLYTEKLMPAVTEGAEKAGRTVADVDTMIEIKLSYE
TDYDLALQNTRFWAPLSLTPEQKHSISDPIEMEAAADALPIEQIAKRWIVGTDPDDVVSR
IEPYIDAGMRHLVFHAPGHDQSRFLELFARDLAPRLRKLG
>A0A0Q8W8X79ACTN
--MELRIGYKASAEQFAPRELVELGVLAEEAGMDTAVVSDHFQPWRHEGGHAPFSLAWLT
AVGERTSSIRLGTSVMTPTFRYNPAVVAQAFATMGCLYPDRILLGVGTGEALNEAASGF-
-VGEWPEFKERFARLREAVDLMRQLWTGER-VDFEGDHYRTVGASIYDVPDGGIPVYVAA
GGPVVARYAGRKGDGMICTSGKGMELYEDKLMPALAEGAEKAGRSADDIDRMIEIKVSYD
PDPQVALENTRFWAPLSLTAEQKHSIDDPMEMEAAADALPIEQVAKRWIVSSDPDEVAEL
VKPYTDAGLNHLVLHAPGHDQRRFLELFASDLAPRLRALG
>A0A0Q6VD979ACTN
--MELRIGYKASAEQFAPRELVELGVLAEAAGMDTAVVSDHFQPWRHEGGHAPFSLAWLT
AVGERTSSIRLGTSVMTPTFRYNPAVVAQAFATMGCLYPGRIILGVGTGEALNEAASGF-
-VGEWPEFKERFARLREAVDLMRQLWTGER-VDFEGDHYRTVGASIYDVPDGGVPVYVAA
GGPVVARYAGRKGDGMICTSGKGMDLYEDKLMPALAEGADKAGRSTDDIDRMIEIKVSYD
PDPQVALENTRFWAPLSLTAEQKHSIDDPMEMEAAADALPIEQVAKRWIVSSDPDEVAAL
VKPYVDAGLNHLVLHAPGHDQRRFLELFSTDLAPRLRALG
>G7GY399ACTN
MTQGLKLGFKASAEQFDPRELVEIAVAAEEHGLDSVAVSDHFQPWRHNGGHAPFSLAWMA
AVGERTERVQIGTSVMTPTFRYNPAVIAQAFATMGCLYPGRIMLGVGTGEALNEYATGF-
-TGDWPEFKERFARLREAIGLMRELWTGEK-VDFEGEYYRTQGAFMYDVPEQPIPVYVAA
GGPVVARYAGRAGDGFICTSGKGMELYTEKLIPAVKEGAAKAERDFADIDRMIEIKISYD
PDPELALENTRFWAPLSLTPEQKHSVNSSVEMERLADELPIEQVAKRWIVASDPDEAVEQ
VRQYTDAGLNHLVFHAPGHDQRRFLDNFSRDLAPRLRELV
>K6WAS49ACTN
MAAELKLGFKASAEQFDPRELVEIAVAAEEHGMDSVAVSDHFQPWRHNGGHAPFSIAWMA
AVGERTKRVQIGTSVMTPTFRYNPAVIAQAFASMGCLYPGRIMLGVGTGEALNEYATGF-
-QGEWPEFKERFARLRESITLMRELWTGEQ-VDFDGEYYKTQGAYMYDVPDKPIPVYVAA
GGPVVARYAGRAGDGFICTSGKGADLYQEKLIPAVKEGAEKAGRDFEAIDRMIEIKISYD
PDPELALENTRFWAPLSLTAEQKHSVNSSTEMERLADELPIEQVAKRWIVASDPDEAVEK
VKFYTDCGLNHLVFHAPGHDQRRFLDNFERDLAPRLRKLS
>M3VEA39ACTN
-MAELKLGFKASAEQFDPRELVEIAVAAEEHGLDSVAVSDHFQPWRHNGGHAPFSLAWMA
AVGERTKRVQIGTSVMTPTFRYNPAVIAQAFASMGCMYPGRIMLGVGTGEALNEYATGF-
-QGDWPEFKERFARLREAIRLMRELWTGEE-VNFDGEYYHTQGAYMYDVPEQPIPVYVAA
GGPVVARYAGRAGDGFICTSGKGAELYTEKLIPAVKEGAEKAERDFDAIDKMIEIKISYD
PDPELALENTRFWAPLSLTPEQKHSVNSSVEMERLADELPIEQVAKRWIVSSDPDEAVEK
VKFYIDAGLNHLVFHAPGHDQRRFLENFERDLAPRLRRLA
>G7GIN49ACTN
```

MAQQLKLGFKASAEQFDPRELVEIAVAAEQAGMDSVAVSDHFQPWRHNGGHAPFSLAWMA
AVGERTERVQIGTSVMTPTFRYNPAVIAQAFATMACLYPGRIMLGVGSGEALNEYATGF-
-QGEWPEFKERFARLRESIALMRELWTGEE-VNFDGEYYKTQGAYMYDIPDKPVPVYIAA
GGPVVARYAGRAGDGFICTSGKGAELYTEKLIPAVKEGAEKVGRDTAEIDRMIEIKISYD
PDPELALENTRFWAPLSLTPEQKHSVNSSTEMERLADELPIEQVAKRWIVASDPDEAVEK
VKFYTDAGLNHLVFHAPGHDQRRFLDNFARDLEPRLRNLT
>A0A076MXB7AMYME
--MGLKVGYKASAEQFGPRDLVEYAVRAEELGLDSVMVSDHFLPWRHEGGHAPFALSWMS
AVAERTNRVQIGTSVLTPTFRYNPAVIAQAFATMSLLSGGRVILGVGTGEALNEIAVS--
-GREWPEFKERFARLREAIKLMRELWTSDN-VSFEGEYYTLVNAKIYDRPEQPVPVYVAA
GGPVVAKYAGRAGDGFICTSGKGMELYNDKLMPAVAEGAAAAERDVAGIDKMIEIKMSYD
RDHAKALENTRFWAPLSLTPEQKHSVSSAEEMERLADELPIEQIAKRWIVASDPDEAVAQ
IKPYLDAGLNHLVFHGPGHDQERFLTQFAEDVLPRLRALG
>A0A066TSB69PSEU
--MALKVGYKASAEQFGPRDLVEYAVRAEEVGLDSVWVSDHFLPWRHEGGHAPFALAWMP
AVAERTKRVRIGTSVLTPTFRYNPAVIAQAFATMSLLSNGRVILGVGTGEALNEIAVS--
-GREWPEFKERFARLRESIKLIRELWTSDN-VNFDGEYYQLVNAKIYDRPEQPVPVYVAA
GGPVVAKYAGRSGEGFICTSGKGMDLYTEKLIPAVQEGATAAERDAESIDRTIEIKMSYD
RDAGKALENTRFWAPLSLTAEQKHTVSSAEEMERLADELPIEQVAKRWIVASDPDEAVAQ
IKPYLDAGLNHLVFHGPGHDQERFLTQFSEDVLPKLRALG
>R1FZ499PSEU
---MLKVGYKASAEQFGPRDLVEYAVRAEEVGLDSVWVSDHFLPWRHEGGHAPWALAWMP
AVAERTKRVQIGTSVLTPTFRYNPAVIAQAFATMSLLSGGRVILGVGTGEALNEIAVS--
-GREWPEFKERFARLRESIKLIRELWTSDN-VNFKGDYYELVDAKIYDRPEQPVPVYVAA
GGPVVAKYAGRAGDGFICTSGKGMDLYTEKLMPAVKEGAEAAEKTVEDVDRTIEIKLSYD
RDHEQALENTRFWAPLSLSAEQKHSVSSAEEMERLADELPIDQVAKRWIVASDPDEAVAQ
IKPYLDAGLNHLVFHGPGHDQERFLIQFSEDVLPKLRALG
>FGDAMYMU
---MLKVGYKASAEQFGPRDLVEYAVRAEEVGLDSVWVSDHFLPWRHEGGHAPWALAWMP
AVAERTKRVQIGTSVLTPTFRYNPAVIAQAFATMSLLSNGRVILGVGSGEALNEIAVS--
-GREWPEFKERFARLRESIKLIRELWTSDN-VNFKGDYYELVDAKIYDRPEQPVPVYIAA
GGPVVAKYAGRAGDGFICTSGKGMELYTDKLMPAVKEGAEAAEKTVEDVDRTIEIKLSYD
RDHEKALENTRFWAPLSLSAEQKHSVSSAEEMERLADELPIDQVAKRWIVASDPDEAVAQ
IKPYLDAGLNHLVFHGPGHDQERFLTQFSEDVLPKLRALG
>G0FLU9AMYMS
---MLKVGYKASAEQFGPRDLVEYAVRAEEVGLDSVWVSDHFLPWRHEGGHAPWALAWMP
AVAERTKRVQIGTSVLTPTFRYNPAVIAQAFATMSLLSNGRVILGVGSGEALNEIAVS--
-GREWPEFKERFARLRESIKLIRELWTSDN-VNFKGDYYELVDAKIYDRPEQPVPVYIAA
GGPVVAKYAGRAGDGFICTSGKGMELYTDKLMPAVKEGAEAAEKTVEDVDRTIEIKLSYD
RDHEKALENTRFWAPLSLSAEQKHSVSSAEEMERLADELPIDQVAKRWIVASDPDEAVAQ
IKPYLDAGLNHLVFHGPGHDQERFLTQFSEDVLPKLRALG
>F4CUF7PSEUX
--MALRIGYKASAEQFGPRDLVEYAVRAEEVGLDSVWTSDHFLPWRDTGGHAPFALTWMA
AVGERTSRVQIGTSVLTPTFRYNPAVIAQAFASMALMFDGRVALGVGTGEALNEIAVS--
-GREWPEFKERFARLREAVRLIRALWTEDS-VSVDGDYYTLVDAKIYDRPKEPVPVYIAA
GGPVVAKYAGRSGDGFICTSGKGMDLYTEKLIPAVQEGAAAAERDAAGIDRMIEIKLSYD
RDADQALENCRFWAPLSLTAEQKHSVDSAAEMERLADELPIEQVAKRWIVASTPDEALAQ
ITPYVDAGLTHLVFHGPGHDQERFLSQFAEDVLPRLRELG
>A0A0M5LTF09PSEU
--MDLKIGYKASAEQFGPRDLVEYAVLAEELGLDSVWASDHFLPWRHEGGHAPAALPWMA
AVGERTKRVQIGTSVLTPTFRYNPAVLAQEFATMALLTGNRVALGVGTGEALNEIAVS--
-GREWPEFKERFARLREAVKMMRALWTEES-VSTEGEYYTFVDAMIYDRPEQPVPIYVAA
GGPMVARFAGRFGDGFICTSGKGMELYTDKLIPAVEEGAEKADRDASGIDRMIEIKISYD
RDPDAARENTRFWAPLSLTPEQKNAVDSSREMERLADELPIEQVVKRWIVASDPDEAVAQ
IKPYLDAGLNHLVVHGPGHDQRRFLEQFTTDVAPKLRALA
>A0A132MHI39ACTN
VQMGLKIGYKASAEQFGPRELVEFAVRAEELGLDSVMVSDHFQPWRHNGGHAPFSMSWLS
AVGERTSRIVLGTSVLTPTFRYNPAVVAQAFGTLGCLYPGRVILGIGTGEALNEVAVS--
-RMEWPGFKERFARLREAVELMRALWRDER-VTFEGDYYQTTNATVYDRPPGGIPIYVAA
GGPVVAKYAGRAGDGFICTSGKGMELYTDKLLPAVAEGAAASGRDPDAIERMIEIKLSFD
PDPDLALENTRFWAPLALSAEQKHGLEDPVEMERAGDELSIEQVASRWIVTSDPDEAVAA
IKAYVDAGFNHLVFHGPGHDQFRFLTSFAEQVLPRLRALD
>A0A0F0ESN49MICO
---MLKVGWKASAEQFGPRDLVTYAARAEEVGLDSVFISDHFQPWRHQDGHAPFAMSWLA
AAGERTERIQLGTSVMTPTFRYNPAVVAQAFGTLGALNPGRIVLGIGTGEALNEVAVGAA
-GSPWPEFKERFARLREAVTLMRRLWTEER-VEFEGEYYRTHAATVYDRPEQPIPVYVAA
GGPLVARYAGRSGDGFICTSGKGRELYEDKLLPAVDEGLAKSGRTRDDIDKMIEIKLSYD
RDADQALHNVRFWAPLSLSAEQKHGVDDPVEMERLADELSDEEIAKRWIVTSDPQEAVDA

```
VRQYVDWGFDHVVFHAPGHDQGRFLEQFSADVLPGLRELG
>A0A0A0CBM09CELL
----MRIGYKASAEQFGPRELVDLAVLAEQAGLDSVWVSDHFQPWRHEGGHAPFALAVLA
AAGERTSRVLLGTSVLTPTFRYNPAVLAQAFGTLGVLFPGRVALGVGSGEALNERAVDP-
-ALEWPEFKERFARLRESVELMRALWAGDR-VSFDGAFYATSDATVYDRPSSGLPVYVAA
GGPVVARYAGRVGDGFICTSGKGVELYRDKLMPAVAEGAAAAGRDPSGIDKMIEIKLSYA
ADRATALEACRFWAPLSLTAEQKHGVDDPTEMARLADRLPIEEVAQRWIVATTAEEVVEQ
VRFYRDLGFDHLVFHAPGHDQAAFLESFGNDVLPALRAL-
>A0A0K1F7549MICO
--MAPRIGYKASAEQFAPRDLAGYAILAEELGLDSAFIADHFQPWRHTGGHAPSSIPWLA
HVAARTERILVGTSVMTPTFRYNPAMVAQAFATLACLHPGRIVLGVGTGEALNEIAVGSV
-EGDWPDFKERFARLREAVELMRRLWTEER-VTHEGTYYRTQDATVYDRPESPVPVYVAA
GGPMVARYAGRKGDGFICTSGKGRELYADQLVPAVVEGAAKAGRSADELDRMIEIKLSYD
PDPAAALENCRFWAPLSLTAEQKHSLHDPAEMEAAADALPIEQVARRWIVASTPEEVVEQ
VREYTDLGFDHLVFHGPGHDQERFLRTFAEQVVPALRSLT
>A0A0Q9MG309MICO
TSQRLRIGYKASAEQFDPAGLAGFAVLAEELGLDSVTISDHFQPWRLEGGHAPNSIAWMS
WVLARTERIMLGTSVMTPTFRYNPPVVAQTFATMACLAPGRVMLGVGTGEALNEIAVGSV
TEGEWPEFKERFARLREAVTLMRQLWTESR-VTFEGEYYRTEDAAIYDRPDQPVPVYIAA
GGPMVARYAGRHGDGFICTSGKGEELYRDQLVPAVDEGLDKVGRSHDDIDRMIEIKVSYD
PDPEQALENTRFWAPLSLSAEQKHSIHSPEEMERAADELPIEQVAKRWVVTSKPEDVVEA
LRFYTDLGFDHLVVHGPGHDQERFLRTFTEQVVPGLRELV
>D9VBX09ACTN
RTSGITIGYKASAEQFGPRHLVELAVLAERRGFDSVLVSDHYQPWRHRNGHAPFSMAWLA
AAGERTERVRLGTSVLTATFRYHPAVVAQAFGTLGALCPGRVMLGLGTGEALNEVAVA--
-RMEWPGFEERFARLREAIDLIRRLWTEER-VSFDGEYYRTENATVYDRPSRPVPVYVAA
GGPVVAKYAGRIADGFICTSGKGMELYTEKLQPAVDAGAEQAGREPADVARTIEIKLSYD
TDAEAAAENTRFWAPLSLTADQKHGVSDPLAMERAADELPMSQIASRWIVSSDPDEVVER
IRPYVDAGFTDLVLHAPGHDQARFLELARQDLLPRLRNLG
>D6TUI19CHLR
STSRLKFGYKASAEQFGPRELLDFTVEAEDLGFDSVWISDHFQPWRHTNGHAPFALSWLG
SAAERTKKIVLGTSVLTPTFRYQPAVVAQAFGTLGLMYPGRFILGVGSGESLNEVAVTG-
--MQWPEAKERLARLRESVRLIKQLWQEDM-VTFNGEYYHTLNATIYDKPKQPVPIYIGA
GGPVAAKFAGREGDGFICTSGKGDELYRDKLLPAMEEGARAAGKDPQSVERTIEVKVSFD
TDKDRALKDTRIWAALALPAEDKVSIHDAREMEEKAKSVE-DQAHRRWLVSSDPDEHIEQ
IRPYIELGFTHLIFHAPGDDQSRFLQLYAKEILPRLRQRW
>D6U2M49CHLR
QAHPLKLGYKASAEQFAPRELLNFSVEAEQCGFDSVWISDHFQPWRHTDGHAPQAFAWLG
ALGERTQRVLLGTSVLTPTFRYNPAIVAQAFGTLGVLNPGRMILGVGSGESLNEIAVTG-
--GEWPPAKERLARLRESVELIRRLWSEEL-VTFEGEHYRTRNATIYDKPDQPIPIYISA
GGPVAAKFVGRAGDGFICTSGKGDALYRDQLLPSVAEGAKAAGRDPEQIEKTIEVKVSFD
TDRNRALQDTRIWAALALPAEDKVDIHDAREMEAKAVTV-TDQAHKRWLVSSDPEEHIEQ
IRPYIELGFTHLIFHAPGDDQSRFLQLYAKEILPRLRQRW
>U1MQZ09MICO
----MRYGYKASAEQFSPAELLDLVVLAERVGLDSAFISDHLQPWRHEGGHAPNAVAWLG
MALERTERLVLGTSVLTPTLRYHPAVVAQQFATLGQVHPGRLILGVGTGEALNEQAIGV-
---EYPELKERFARLREAVRVLKRLWTDER-VDFDGDYYSLNGATIYDRPAERIPIYVAG
GGPATTRYAARFADGHICTSGKGDEYYRDTIVASLEEGLAQAERDSAEVDRMIEIKVSYD
RDPDAALEHTRFWAALSLTQEQKHDVHDPLEMQRLADELPIEQVAKRWIVGSDASRVAGE
IAHYAELGFTHLVFHGPGHDQRRFLEQFSEDVLPLLPAR-
>O937349EURY
--MKTQIGYFASLEQYRPMDALEQAIRAEKVGFDSVWVDDHFHPWYHDNAQSAQAWAWMG
AALQATKKVFISTCITCPIMRYNPAIVAQTFATLRQMYPGRVGVAVGAGEAMNEVPVTG-
---EWPSVPVRQDMTVEAVKVMRMLWESDKPVTFKGDYFTLDKAFLYTKPDDEVPLYFSG
MGPKGAKLAGMYGDHLMTVAAAPSTL-KNVTIPKFEEGAREAGKDPSKMEHAMLIWYSVD
PDYDKAVEALRFWAGCLVPSMFKYKVYDPKEVQLHANLVHCDTIKENYMCATDAEEMIKE
IERFKEAGINHFCLGNSSPDVNFGIDIF-KEVIPAVRD--
```

Data S3. Rooted tree of F_{420}-dehydrogenases employed for ASR (newick format)


```
(((E9V3D19ACTN:0.00639,A0A0F2G5349ACTN:0.01511):0.20531,(A0A010YY079ACTN:0.394
37,(((R7XUN79ACTN:0.25932,A0A021VMU49CELL:0.25117):0.09788,((A0A117MS219ACTN:0
.05976,A0A0A6UL69ACTUT:0.05533):0.07244,(U5W9H09ACTN:0.08934,R4LRU99ACTN:0.045
83):0.07137):0.15431):0.05249,(A0A0Q6R9Q99MICO:0.43851,(((D6TUI19CHLR:0.15076,
D6U2M49CHLR:0.24145):0.47174,(U1MQZ09MICO:0.71848,(D9VBX09ACTN:0.41855,((A0A0F
0ESN49MICO:0.37055,((A0A0K1F7549MICO:0.19835,A0A0Q9MG309MICO:0.26778):0.23875,
((A0A0M5LTF09PSEU:0.18278,(F4CUF7PSEUX:0.16131,(A0A076MXB7AMYME:0.05243,(A0A06
6TSB69PSEU:0.04702,(R1FZ499PSEU:0.01422,(FGDAMYMU:0.00000,G0FLU9AMYMS:0.00000)
:0.01603):0.07329):0.06144):0.07883):0.05052):0.16698,((G7GY399ACTN:0.04626,(M
3VEA39ACTN:0.03811,(K6WAS49ACTN:0.03526,G7GIN49ACTN:0.09315):0.04997):0.05739)
:0.13709,(((A0A076EZM4RHOOP:0.10408,((W4A0K4RHORH:0.00846,(A0A059MIF19NOCA:0.0
0459,(W3ZYN4RHORH:0.00432,A0A059MM579NOCA:0.01705):0.00832):0.00451):0.08825,(
M2WXX69NOCA:0.04984,A0A0K2YIJ99NOCA:0.03627):0.02422):0.02245):0.03511,((A0A0Q
8W8X79ACTN:0.01329,A0A0Q6VD979ACTN:0.03507):0.24644,((FGDNOCFA:0.00000,A0A0H5P
GI5NOCFR:0.00420):0.05518,((A0A0C1AY679NOCA:0.00000,(A0A034UHX49NOCA:0.00000,K
0FE219NOCA:0.00000):0.00000):0.05162,(A0A0B8N8Q49NOCA:0.04972,(A0A034TYZ89NOCA
:0.00000,(A0A0C1CMG39NOCA:0.00000,K0F4H79NOCA:0.00000):0.00410):0.25418):0.042
13):0.01993):0.07916):0.01519):0.01840,((K5BH82MYCHD:0.06182,((Q9XC09MYCPH:0.0
1151,(A0A100X7359MYCO:0.00605,A0A117K3969MYCO:0.01716):0.06328):0.00977,((A0A0
B2YM229MYCO:0.06038,(A0A0D1L1H49MYCO:0.06747,(A0A024JU469MYCO:0.05160,(A0A0J6W
3L29MYCO:0.02807,(L0IPW19MYCO:0.03267,((A0A132SJE89MYCO:0.02351,(A1T2Y6MYCVP:0
.01782,A0A024QK85MYCNE:0.02229):0.00000):0.00459,(FGDMYCGI:0.02047,((V5X8A3MYC
NE:0.02274,A0A0Q8ZNK99MYCO:0.01439):0.02114,(A0A0G3IEC49MYCO:0.02235,(I4BDD3MY
CCN:0.04531,(A0A127RDL8MYCSI:0.01159,J4SKB89MYCO:0.03477):0.03698):0.02305):0.
01905):0.00841):0.00853):0.00834):0.00459):0.01935):0.01488):0.01987):0.02447,
(X5L6I09MYCO:0.05151,(A0A0K0WZZ69MYCO:0.04745,(A0A0N9X869MYCFO:0.00000,FGDMYCF
O:0.01448):0.03894):0.01421):0.02386):0.02030):0.00825):0.03524,(A0A164E6D4MYC
KA:0.08097,((A0A0U0WAL19MYCO:0.04067,(A0A0U1D7K19MYCO:0.03015,(A0A0J8UVG79MYCO
:0.01739,I0REJ9MYCXE:0.01833):0.07800):0.02062):0.00000,(A0A0Q2LLI1MYCGO:0.084
63,((I2AK149MYCO:0.00402,(S4ZLU29MYCO:0.00000,A0A081I3N89MYCO:0.00000):0.00471
):0.03200,((V7K5R9MYCAV:0.00429,V7L3J0MYCAV:0.00000):0.00433,((X8B6F8MYCAV:0.0
0000,(X7UM259MYCO:0.00000,T2GXG4MYCHT:0.00432):0.00431):0.00000,(V7KG82MYCPC:0
.00000,(V7IYU4MYCAV:0.00000,(V7MV86MYCAV:0.00000,(FGDMYCA1:0.00000,(A0A049DNN8
MYCAV:0.00000,(A0A0E2WGP5MYCAV:0.00000,(Q6R2X5MYCPC:0.00000,(V7NPB8MYCAV:0.000
00,A0A0E2WNU7MYCAV:0.00000):0.00000):0.00000):0.00000):0.00000):0.00000):0.000
00):0.00000):0.00000):0.00000):0.03321):0.01272):0.01024):0.02761):0.02696):0.
09027):0.08905):0.14152):0.06046):0.06876):0.02427,(A0A132MHI39ACTN:0.29203,A0
A0A0CBM09CELL:0.45601):0.04983):0.04914):0.12515):0.15913):0.22650,(FGDNAKMY:0
.30091,((A0A0Q8LV009MICO:0.07891,A0A0Q8AXV79MICO:0.08962):0.22615,(A0A163G2939
MICO:0.05075,(A0A147EU64MICTE:0.00139,(A0A147F4G7MICTE:0.01269,((FGDMICTS:0.00
000,A0A0Q5KAR99MICO:0.06370):0.00478,(A0A074TY249MICO:0.02261,A0A0Q6ECN29MICO:
0.03119):0.02632):0.00000):0.02070):0.08152):0.17631):0.19088):0.05792):0.0482
0):0.06931):0.13872):0.18139):1.51579,O937349EURY:2.94964);
```
